# New clues for the role of cerebellum in schizophrenia and the associated cognitive impairment

**DOI:** 10.3389/fncel.2024.1386583

**Published:** 2024-05-10

**Authors:** Pawan Faris, Doris Pischedda, Fulvia Palesi, Egidio D’Angelo

**Affiliations:** ^1^Department of Brain and Behavioral Sciences, University of Pavia, Pavia, Italy; ^2^Digital Neuroscience Center, IRCCS Mondino Foundation, Pavia, Italy

**Keywords:** cerebellum, schizophrenia, cognitive impairment, cerebellar neurotransmitters, cerebellar connectivity

## Abstract

Schizophrenia (SZ) is a complex neuropsychiatric disorder associated with severe cognitive dysfunction. Although research has mainly focused on forebrain abnormalities, emerging results support the involvement of the cerebellum in SZ physiopathology, particularly in Cognitive Impairment Associated with SZ (CIAS). Besides its role in motor learning and control, the cerebellum is implicated in cognition and emotion. Recent research suggests that structural and functional changes in the cerebellum are linked to deficits in various cognitive domains including attention, working memory, and decision-making. Moreover, cerebellar dysfunction is related to altered cerebellar circuit activities and connectivity with brain regions associated with cognitive processing. This review delves into the role of the cerebellum in CIAS. We initially consider the major forebrain alterations in CIAS, addressing impairments in neurotransmitter systems, synaptic plasticity, and connectivity. We then focus on recent findings showing that several mechanisms are also altered in the cerebellum and that cerebellar communication with the forebrain is impaired. This evidence implicates the cerebellum as a key component of circuits underpinning CIAS physiopathology. Further studies addressing cerebellar involvement in SZ and CIAS are warranted and might open new perspectives toward understanding the physiopathology and effective treatment of these disorders.

## 1 Introduction

Schizophrenia (SZ) is a complex neuropsychiatric syndrome affecting approximately 1% of the population worldwide. Although it is considered a low-prevalence illness, the burden of SZ is substantial and ranks among the top 10 causes of disability globally (Owen et al., 2016; Charlson et al., 2018; Marder and Cannon, 2019). SZ was traditionally classified into several categories, such as paranoid, hebephrenic, undifferentiated, residual, catatonic, and simple. In 2013, a significant change was made with the release of the fifth edition of the Diagnostic and Statistical Manual of Mental Disorders (DSM-5). Following debate, DSM-5 abandoned the traditional subtypes due to several factors and controversies concerning their clinical utility and reliability. The field shifted away from subtyping toward a broader diagnostic framework called *“schizophrenia spectrum disorder”* due to the need for a more comprehensive dimensional approach to understanding the heterogeneity of SZ. This spectrum reflects the broader conceptual framework outlined in the DSM-5, which recognizes a spectrum of related conditions beyond classical SZ, includes diagnoses such as schizoaffective disorder, schizophreniform disorder, and others, acknowledging the diversity within psychotic disorders. This allows healthcare professionals to diagnose the condition based on the severity of symptoms (American Psychiatric Association, 2013). Symptoms typically appear during the late teenage or early adulthood, mainly among men, while it becomes prevalent in women from age 40 onwards (Charlson et al., 2018; Velligan and Rao, 2023). The onset of SZ during early neurodevelopment establishes it as a neurodevelopmental disorder (Fatemi and Folsom, 2009). The symptoms, course, prognosis, and treatment efficacy vary from patient to patient. It encompasses a range of symptoms, including *hallucinations and delusions (positive), lack of emotion, joy, and motivation (negative), and impaired memory, attention, learning, and decision-making (cognitive)* (Patel et al., 2014; Queirós et al., 2019). Among these, the “*Cognitive Impairment Associated with SZ”* (CIAS) is core, accounting for much of the impaired functioning associated with the disorder not responsive to existing therapies (McCutcheon et al., 2023). This may result in higher rates of co-incidence of medical and/or mental illnesses, such as substance abuse, mainly alcohol and cannabis consumption, with prevalence rates up to 41.7% (Hunt et al., 2018), and more likely to get health complications, including cardiovascular disorders (Nielsen et al., 2021), diabetes (Mamakou et al., 2018), immune-related disorders (Pouget et al., 2019), endocrine dysfunctions (Misiak et al., 2021), and respiratory diseases (Suetani et al., 2021). Consequently, SZ patients show higher mortality rates than healthy individuals (Correll et al., 2022). Given the complex nature and wide range of variables involved, the etiology of SZ is multifaceted and requires extensive investigation and an integrated approach. Nonetheless, recent neurobiological research has implicated several factors in the development of SZ (Orsolini et al., 2022), including:

*(i) Genetic:* SZ is a *highly heritable disease*, with several genetic alterations implicated in its onset and development, including copy number variants (CNVs), genetic mutations, risk genes, gene polymorphism, and single nucleotide polymorphism, which mainly affect brain functionality during pre-pubertal and pubertal age (DeLisi, 2022; Owen et al., 2023). Family and twin studies show an incidence risk of around 80% (Sullivan et al., 2003; Lichtenstein et al., 2009), while the remaining 20% is attributed to non-heritable factors, including environmental, stochastic, and *de novo* mutations risk factors. It is worth noting that the 22q11.2 deletion (a type of CNV) showed the highest effective size among SZ patients (Owen et al., 2023). The expression of specific gene alleles of the major histocompatibility complex (MHC) has been related to alterations in white matter microstructure within tracts innervating the frontal lobe (Emily Simmonds et al., 2023), particularly on chromosome 6 and 19, influencing axonal density in tracts connecting to the frontal lobe. This suggests that alterations in axonal packing, driven by MHC risk alleles, might represent a neurobiological mechanism in SZ. Family cohorts, linkage studies, and genome-wide association studies (GWAS) led to identifying multiple loci-related SZ risks (Allen et al., 2008; DeLisi, 2009; Ripke et al., 2020).

*(ii) Epigenetic:* Epigenetic alterations involve modifications to gene expression influenced by external cues, and are hypothesized to act as a mediator of environmental risk factors (see iii) involved in SZ pathophysiology (Rivollier et al., 2014; Chen et al., 2021). Epigenome-wide association study approaches led to the discovery of genetic loci that undergo differential epigenetic regulation (Perzel Mandell et al., 2021). *Neurodevelopmental dysfunction*, which is hypothesized as a significant contributor to SZ beyond genetic influences, was initially proposed by Weinberger, who posits that genetic predisposition and environmental insults during gestation alter the neurodevelopmental process and are latent until the maturational changes of adolescent exposure to earlier neurodevelopmental abnormalities (Weinberger, 1987). These findings underscore the complexity of SZ etiology, wherein both genetic and environmental factors interact to shape neurodevelopmental trajectories and contribute to structural and functional alterations in different brain regions. Sustained brain development into young adulthood emphasizes vulnerability until typical SZ onset. *The “two-hit” and “multiple hits” hypotheses* propose that the chance of developing SZ increases with exposure to several risk factors that alter essential processes during ongoing development (Davis et al., 2016). A comprehensive analysis in a cohort of 381 SZ patients showed a significant contribution of DNA methylation alterations to the phenotypic diversity including cognitive deficits (Kiltschewskij et al., 2023). These factors may lead to structural/functional alterations in different brain regions.

*(iii) Environmental*: Several environmental factors, including abnormal fetal development and low birth weight, pregnancy-related diabetes, preeclampsia, other birthing complications, maternal malnutrition and vitamin D deficiency during pregnancy, winter births (which are associated with a 10% higher relative risk), social environment, urban residence, childhood trauma or stress are implicated in the development of SZ (Jones, 2013; Löhrs and Hasan, 2019; Crossley et al., 2021; King et al., 2023). Moreover, immune dysfunctions and neuro-inflammatory processes seem to play a role in SZ pathogenesis. Studies evidenced the involvement of neuro-inflammation (Müller, 2018) and autoinflammation in SZ (Delunardo et al., 2016) but microglia activation has not been confirmed by PET studies in humans (Marques et al., 2019). Compared with healthy individuals, SZ patients have an older brain for their chronological age and have the most pronounced acceleration of brain aging based on the model of frontal features (Kaufmann et al., 2019). Thus, early aging negatively affects the brain volume causing an age gap that is considered a potential biomarker of SZ (Man et al., 2021). Moreover, structural and functional abnormalities in some brain regions potentially cause deviations in the brain aging trajectory (Ballester et al., 2023). On the contrary, data from 26 cohorts suggested that advanced structural brain aging among SZ is not associated with specific clinical characteristics (Constantinides et al., 2023).

Several therapeutic strategies exist to reduce the symptoms, including antipsychotics, psychosocial interventions, electroconvulsive therapy, and alternative and complementary therapies (Correll et al., 2023). However, each approach has significant limitations (Khandaker et al., 2015; Stępnicki et al., 2018). Since the existing therapies may only be effective for 50% of patients, the estimated life expectancy of SZ is 15–20 years shorter than that of the general population (Correll et al., 2022), and it mostly addresses positive symptoms rather than negative and cognitive symptoms and causes multiple neurological and metabolic complications (Stępnicki et al., 2018; Robbins, 2019). Therefore, understanding SZ’s mechanisms and alterations is crucial for developing novel, mechanism-based therapies.

Brain imaging techniques have shown abnormalities in different brain regions (Karlsgodt et al., 2010; Zhao et al., 2018; Khalil et al., 2022; Sone et al., 2022). The alterations include *reduction in brain volume, particularly in frontal and temporal regions, abnormal connectivity between brain nodes, and altered neurotransmitter activity* (Wright et al., 2000; Dean, 2002; Cannon et al., 2003; Karlsgodt et al., 2010; McCutcheon et al., 2020). Decades ago, Andreasen and colleagues, developed a model that implicates the connectivity among brain nodes located in prefrontal regions, thalamic nuclei, and cerebellum, suggesting that disruption in this circuitry produces “*cognitive dysmetria*” (Andreasen et al., 1998), encompasses difficulty in prioritizing, processing, coordinating, and responding to information. Subsequently, the cerebellum started to attract attention due to its potential role in the SZ physiopathology.

The cerebellum, historically known to control movement and motor coordination, gaining importance also for its involvement in different cognitive, affective, and social functions (D'Angelo and Casali, 2012; Schmahmann, 2019; Jacobi et al., 2021; Supplementary material). Failure of cerebellar functioning determines the so-called *cerebellar cognitive affective syndrome* (CCAS) (Schmahmann and Sherman, 1998) in addition to ataxia. Furthermore, studies exploring the organization of cerebro-cortical pathways have shown intricate connections between the cerebellum and various regions of the brain involved in high cognitive functions (Palesi et al., 2015, 2017; Schmahmann, 2019; Jacobi et al., 2021). The concept of the *universal cerebellar transform* suggests that the cerebellum expresses a fundamental computational capability that extends beyond motor processing (D'Angelo and Casali, 2012). This notion is in line with the *dysmetria of thought* theory, proposing that the cerebellum participates in cognitive operations by fine-tuning the timing and coordination of mental processes (Andreasen and Pierson, 2008; Yeganeh-Doost et al., 2011; Bernard and Mittal, 2015; Cao and Cannon, 2019; Schmahmann, 2019). Growing evidence suggests that cerebellar abnormalities play a central role in the pathophysiology of SZ (Andreasen and Pierson, 2008; Yeganeh-Doost et al., 2011; Bernard and Mittal, 2015; Cao and Cannon, 2019) by influencing cortical processing (Andreasen and Pierson, 2008; Yeganeh-Doost et al., 2011; D'Mello et al., 2015; Mapelli J. et al., 2022).

**Figure 1:**
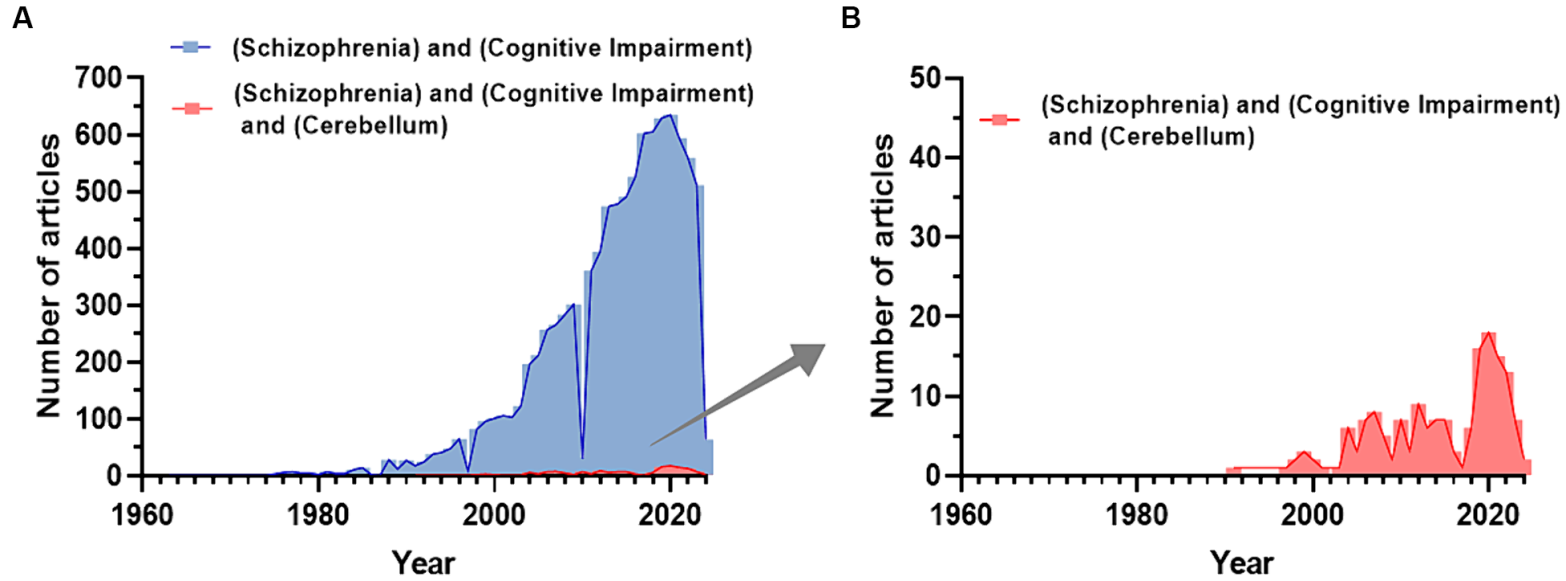
Trend of publications about CIAS on PubMed. **(A)** Number of publications related to the keywords “Schizophrenia and Cognitive Impairment” extracted from PubMed. **(B)** The number of publications related to the keywords “Schizophrenia and Cognitive Impairment and Cerebellum” extracted from PubMed. Publications including the cerebellum start to appear with a delay of about 30 years and reach a maximum of about 1/30 of the total publications on SZ and Cognitive impairment (Figures are created with the GraphPad prism 8).

In the last 50 years, the number of PubMed articles including the terms “schizophrenia” and “cognitive impairment” has increased (Figure 1A), while adding “cerebellum” it remains notably smaller (Figure 1B). This cannot solely be due to the time gap (the cerebellum hypothesis in CIAS was introduced 25 years ago) highlighting the need for further investigating the field. In this review, we first summarize recent literature about brain abnormalities in CIAS and then explore the involvement of cerebellum, emphasizing structural and functional abnormalities, along with alterations in neurotransmitter systems and connectivity.

## 2 Neural dysfunction in cognitive impairment associated with SZ (CIAS): the classical view

Factor analyses of the Measurement and Treatment to Improve Cognition in SZ test battery identified seven cognitive domains: (1) processing speed, (2) attention, (3) working memory, (4) verbal learning and memory, (5) visual learning and memory, (6) reasoning, and (7) social cognition and executive functions (Nuechterlein et al., 2004). Eventually, dimensionality reduction suggests that these seven domains can be reduced to the parent domains of *processing speed, attention/working memory, and learning* (Burton et al., 2013) bearing relevant cerebellar implications (see below). Among these, processing speed is the most affected domain in SZ. However, it is associated with antipsychotic treatments, and the severity of the impairment does not differ from that observed in verbal and working memory (Knowles et al., 2010; Yeganeh-Doost et al., 2011; D'Angelo and Casali, 2012; Fatouros-Bergman et al., 2014). Considering the time course of CIAS, the overall cognitive impairment is detectable during childhood, while the severity of verbal and nonverbal deficits increases throughout the first two decades of life (Mollon et al., 2018). These deficits can be observed in the first episode and are more severe among the clinical high-risk group. However, cognitive symptoms may manifest before the stabilization of psychotic symptoms during adolescence, and evidence suggests they could even be evident before the onset of psychosis. Notably, the decline of cognitive processes throughout the illness is considered a defining feature of SZ (Hedges et al., 2022). Indeed, most cognitive decline over two decades post-hospitalization often exceeds normal aging. This underscores the importance of focusing on cognition as a therapeutic target during later stages of psychotic illness (Fett et al., 2020). As cognitive shortages are present before the prodromal period and persist throughout the development of the disease, they could be a potent biomarker and target for early detection and prevention. The severity of cognitive impairment is greater in SZ compared to other psychiatric disorders. A meta-analysis suggested more severe cognitive symptoms, particularly in attention and social cognition, among SZ patients compared to bipolar disorder (Li et al., 2020).

Twin and GWAS studies showed a strong negative correlation between liability for SZ and cognitive function (Toulopoulou et al., 2007; Davies et al., 2018; Savage et al., 2018). Additionally, several deficits in the ability to detect sarcasm were observed among mono and heterozygous twin groups as compared to healthy co-twin. However, impairments were also observed in the unaffected homozygous co-twins, indicating that socio-cognitive deficits could be a genetic vulnerability indicator of the illness. The socio-cognitive decline was associated with lower intelligence and higher levels of psychopathology among SZ patients (Lemvigh et al., 2022). Several genes have been correlated with CIAS, due to their implication in modeling and shaping neuronal plasticity, including *DISC1*, *NRG1*, *AKT1,* and *DTNBP1*, which influence cognitive abilities in SZ (Tripathi et al., 2018). These alterations were detected at both cellular (neuron and glia) and circuit levels (Millan et al., 2012). Nevertheless, the genetic underpinnings of cognitive abilities do not imply a direct link. For instance, when the individual has less access to educational opportunities, the phenotype-associated alleles might negatively correlate with cognitive capacity. Therefore, it is fundamental to consider environmental influences too. Aberrant communication between brain regions and processing in cortical columns are the core pathology of CIAS and are thought to involve alterations in synaptic plasticity and network connectivity. Alterations in gamma band activity were correlated with SZ susceptibility, indicating that shifts in synaptic function and neuronal firing patterns are of pathophysiological relevance rather than consequences of this disorder (Dimitriadis et al., 2021). Alpha and beta band activity was correlated with disrupted temporal connectivity in (para) limbic areas and associated with reduced signal memory and higher variability across time in SZ patients (Alamian et al., 2020). Several neuronal-based alterations seem to contribute to CIAS, including abnormalities in neurotransmitter systems, structural/functional changes (Karlsgodt et al., 2010; Zhao et al., 2018; Sone et al., 2022), impaired synaptic plasticity, and deviations in neural oscillations. Consequently, multiple neurophysiological and neurochemical models were proposed in CIAS (Javitt, 2023).

In this section, we present relevant studies on neuromodulator abnormalities and related hypotheses in CIAS, focusing on cerebral regions following the classical view. The potential role of the cerebellum will be discussed in the subsequent section of this review.

### 2.1 Abnormal neurotransmitter systems

**Figure 2:**
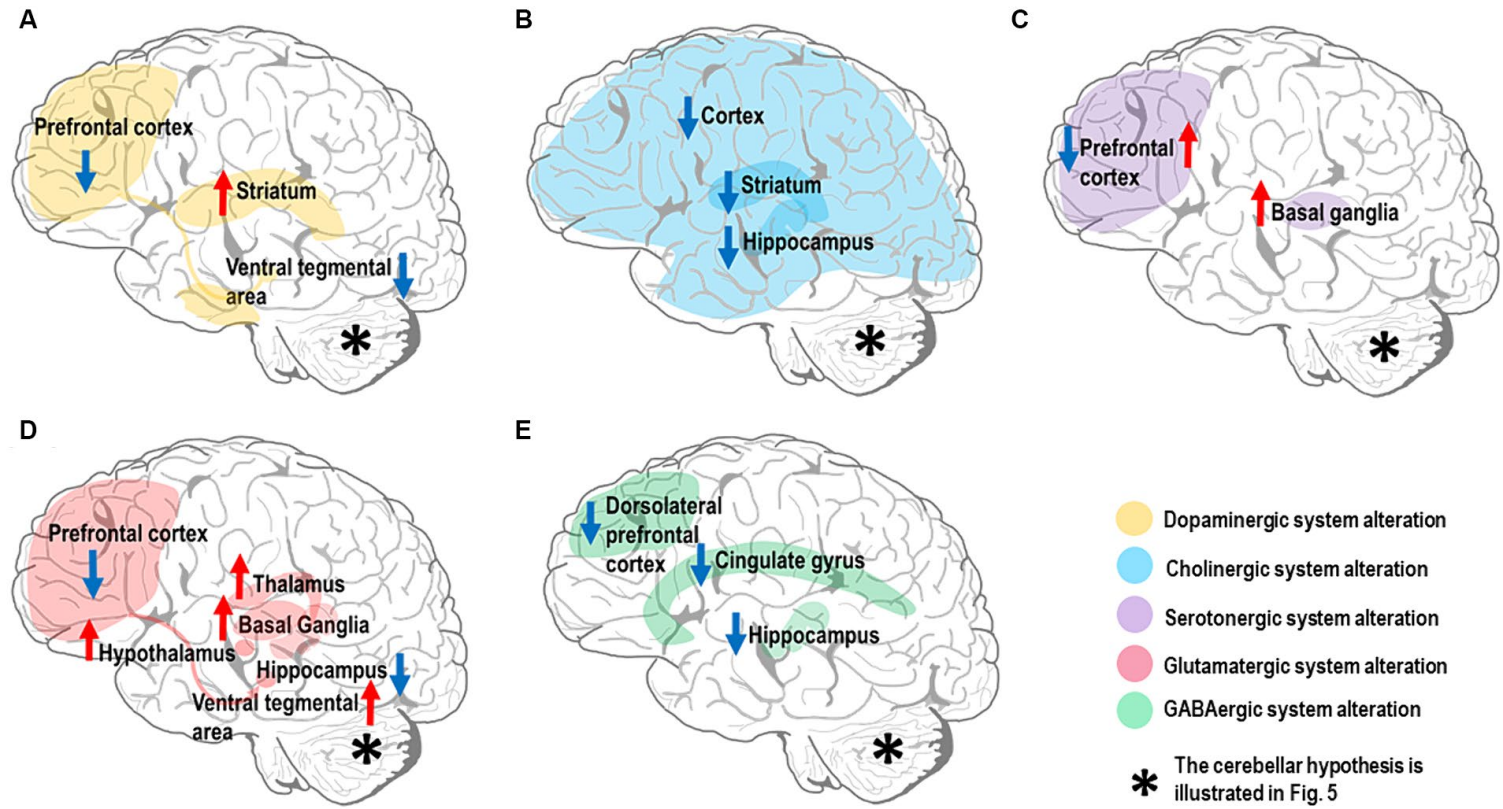
Neurotransmitter alterations in the SZ brain. The most affected brain regions are shown in different colors depending on the neurotransmitter system they belong to. Hyperfunctioning and hypofunctioning regions are identified with red and blue arrows, respectively. Note that the cerebellar neurotransmitter hypothesis is illustrated in Figure 5. **(A)** Dopamine system: there is evidence for dopamine hypofunction in prefrontal cortex and ventral tegmental area (VTA) (mesocortical pathway). In contrast, there is dopamine hyperfunction in the striatum (mesolimbic pathway). **(B)** Cholinergic system: there is evidence for alteration of the cholinergic system in multiple brain regions, including downregulation of muscarinic receptors (M1 and M4) in both cerebral cortex and striatum, along with reduction of cholinergic signaling in the hippocampus. **(C)** Serotonergic system: there is evidence for high serotonin levels and low 5-HT2 receptor density in the prefrontal cortex, while the serotonin level and its metabolites are elevated in basal ganglia. **(D)** Glutamatergic system: there is evidence for multiple alterations in the brain glutamatergic system; NMDARs in prefrontal cortex and iGlutRs in hippocampus are hypofunctional, while excessive glutamate release is detected in basal ganglia, thalamus, hypothalamus, and VTA. **(E)** GABAergic system: there is evidence for decreased GABA concentration in the cingulate gyrus, particularly dorsal anterior cingulate cortex, and dorsolateral prefrontal cortical GABA-neurons; a low α5-GABAARs expression level is found in the hippocampus.

Several neurotransmitters have been implicated in SZ pathogenesis, but whether their alterations are causative, compensatory, or simply consequential remains unclear. SZ patients have altered levels and activity of neurotransmitter systems, especially dopamine, acetylcholine (Ach), serotonin, glutamate, and GABA (Mandal et al., 2022), Impacting multiple brain circuits through disruption of the Excitatory/Inhibitory (E/I) balance (Liu et al., 2021). Notably, dysfunctions primarily involve the alteration of dopaminergic control. Nevertheless, other neurotransmitter systems appear altered and implicated in CIAS. Here, we illustrate the relevant neurotransmitter hypotheses related to CIAS, particularly those implicated in the forebrain and midbrain regions (Figure 2) and set the basis for SZ physiopathology and pharmacotherapy. Issues related to the cerebellum are considered in the next section.

#### 2.1.1 Neurotransmitter models

Dopamine plays a central role in SZ pathophysiology (Howes and Kapur, 2009) as shown by rich experimental evidence (Simpson et al., 2010; McCutcheon et al., 2019, 2020; Selten and Ormel, 2023). Current findings underscore dopamine’s central role in CIAS (Slifstein et al., 2015; Simpson and Kellendonk, 2017). The dopamine hypothesis was developed post-hoc to account for the serendipitous discovery of the anti-psychotic effect of some dopaminergic drugs (Carlsson and Lindqvist, 1963; Creese et al., 1996), while genetic support is limited (Edwards et al., 2016). The dopaminergic hypothesis can be summarized as the combination of two main effects: (1) excessive dopamine activity in the mesolimbic pathway (Simpson et al., 2010; Elert, 2014; McCutcheon et al., 2019), specifically in the striatum, disrupts the neurotransmitter balance impairing the functioning of other brain regions involved in cognitive processing; (2) reduced dopamine activity in the mesocortical pathway, connecting the ventral tegmental area (VTA) to the prefrontal cortex (PFC), is linked to both negative symptoms and CIAS (Brisch et al., 2014; Elert, 2014; Slifstein et al., 2015; Simpson and Kellendonk, 2017; Figure 2A). The dual dopamine hypothesis (Elert, 2014; McCutcheon et al., 2020) also opens an issue for the functioning of dopaminergic antipsychotics, whose efficacy is largely linked to their affinity for the D2R distributed both in cortical and subcortical regions (Toda and Abi-Dargham, 2007; Howes et al., 2012; de Bartolomeis et al., 2023). Although the dopamine hypothesis is central to SZ, its dysregulation in the striatum and cerebral cortex is just one aspect of the complex pathophysiology of SZ. The cerebellum, whose dopaminergic system is starting to unveil, is also probably key to CIAS, as explained below.

Another relevant system for SZ is the cholinergic one. Ach plays a vital role in cognitive functions, and pharmacological manipulation targeting the Ach system influences attention, episodic, working, and spatial memories (Newman et al., 2012). Several experimental results converge toward the hypothesis of *cholinergic hypofunctioning* (Figure 2B) in SZ. Indeed, notable changes in the cholinergic system linked to CIAS, including (1) *reduced cholinergic activity*, (2) *altered receptor functions* (especially M1 and M4 mAChRs) (Crook, Tomaskovic-Crook et al., 2000, Carruthers et al., 2015), and (3) *disrupted cholinergic-dopaminergic interactions* (Foster et al., 2021). Although direct evidence on cerebellar cholinergic alterations in CIAS is scarce, its investigation could open new potential avenues.

Another neurotransmitter implicated in CIAS is serotonin or 5-hydroxytryptamine (5-HT) (Eggers, 2013). Extensive evidence points to *serotonergic hypofunctioning*, suggesting that targeting the serotonin system might provide a viable treatment for CIAS. Thus, a comprehensive understanding of the mechanisms involved is crucial for designing effective treatments (Figure 2C). The conflicting findings on serotonin alterations, *high level* (Zhang et al., 2011), *low 5-HT2 receptor density*, and *altered enzyme activity* underscore the complexity of its role in CIAS, suggesting potential treatments through serotonin system modulation. However, there are still gaps in understanding broader neural circuits beyond the forebrain and midbrain regions, crucial for addressing cognitive deficits. Thus, considering the serotonergic system in cerebellum (Oostland and van Hooft, 2013) and its influence on other neurotransmitters, consequently, cognitive processes, might further reveal how serotonin dysfunction interacts with CIAS (Section 3.2.3).

The incomplete effectiveness of current antipsychotics suggests that alterations in these systems do not account for most of the negative and cognitive symptoms. Therefore, a glutamatergic model of SZ has been proposed. Elert attributed altered concentrations of dopamine in different brain regions to glutamate dysregulation (Elert, 2014), specifically, glutamate receptors in SZ patients are compromised preventing glutamate from binding to them and dysregulating the function of GABAergic inhibitory interneurons. The lack of inhibition, eventually, causes excessive dopamine in the nucleus accumbens, resulting in positive symptoms of SZ, and reduced dopamine concentration in PFC, leading to negative symptoms (Elert, 2014) (please note that, in Elert’s work, cognitive symptoms are considered together with the negative ones). Extensive evidence, combined with preclinical findings, supports the notion that *alterations in the glutamatergic system,* including neurotransmitter and transporter high levels in different brain regions (Matute et al., 2005; Merritt et al., 2019, 2021; Adams et al., 2022) and reduced NMDAR function (NMDAR hypofunction) (Gonzalez-Burgos and Lewis, 2012; Moghaddam and Javitt, 2012), *play a crucial role in the development of CIAS, thus placing the glutamate system dysfunction at the core of SZ* (Swanton, 2020; Figure 2D). Experimental evidence points also to GABAergic system alteration in SZ (Fatemi and Folsom, 2015). Specifically, *Glutamic Acid Decarboxylase 67 (GAD67) downregulation* (Fatemi et al., 2005; Gonzalez-Burgos and Lewis, 2012; Fujihara, 2023), *low GABA level* (Nakahara et al., 2022), *receptor hypofunction* (Marques et al., 2021) *and downregulation*, and *transmission deficits* are linked to CIAS (Dienel et al., 2023), emphasizing the potential of targeting the GABAergic system for improving CIAS. For a review of the glutamatergic and GABAergic hypothesis together see Yeganeh-Doost et al. (2011). Given the fundamental role of the glutamatergic and GABAergic systems of cerebellum, these will be considered below for their potential contribution to CIAS (Sections 3.2.4, 3.2.5).

Based on glutamatergic and GABAergic alterations, an *altered E/I balance* can eventually explain CIAS (Jelen et al., 2019, Liu et al., 2021, Gawande et al., 2023). Restoring the E/I balance and addressing abnormalities in glutamatergic and GABAergic neurotransmission may offer a potential target to improve cognitive function. In particular, *the cerebellar E/I balance, potentially involved in* var*ious cognitive processes* (Sections 1.2, 1.3 in Supplementary material), is a strong candidate to explore CIAS.

#### 2.1.2 Pharmacological implications

Pharmacologically, multiple strategies are exploited to attack the neurotransmitter systems from different sites to ameliorate SZ positive and negative symptoms. The main drugs used are called typical (e.g., haloperidol and chlorpromazine) or atypical (e.g., olanzapine and risperidone), depending on whether they act on the dopaminergic system or also/exclusively on the others. Typical drugs are dopaminergic inhibitors, mostly acting on D2R, ameliorating the positive symptoms but often worsening the negative ones. Given their poor selectivity, these drugs also lead to side effects, including extrapyramidal disturbances, hyperprolactinemia, cognitive decline (Li et al., 2016; Orzelska-Górka et al., 2022), sedation, and cardiovascular issues (Stępnicki et al., 2018). Atypical antipsychotics, instead, address both positive and negative symptoms by affecting various other receptors and have fewer side effects. This is potentially due to their lower D2R affinity or to their preference for mesolimbic over nigrostriatal pathway receptors (Stępnicki et al., 2018; Orzelska-Górka et al., 2022). Consequently, attempts to address cognitive and negative symptoms are growing, rather than exclusively targeting D2R in the dopaminergic system that mainly ameliorates positive symptoms. While atypical drugs might rescue negative symptoms, their primary efficacy remains in targeting positive ones. We argue that recent knowledge on cerebellar physiology will allow for considering new avenues for antipsychotic drug actions and neuromodulation (e.g., transcranial magnetic stimulation, TMS) in CIAS (Section 4.2).

### 2.2 Abnormal synaptic plasticity

Synaptic plasticity is the ability of synapses, connections between neurons, to endure structural and functional changes in response to stimuli. Short-term changes transiently affect local dynamics of neurotransmitter release and postsynaptic receptor activation, while long-term changes involve biochemical modifications of membrane receptors and ionic channels along with cytoplasmic and nuclear gene regulation, modifying the expression of membrane proteins and the formation/pruning of synapses. Long-term changes occur in the form of either long-term potentiation (LTP) or long-term synaptic depression (LTD). In most cases, these changes involve AMPA and NMDA receptors (Citri and Malenka, 2008), a fact particularly relevant to SZ, in which NMDARs seem to play a central role (Krystal et al., 1994; Verma and Moghaddam, 1996; Catts et al., 2016). In SZ, large-scale gene expression analyses showed minor but significant differences in genes associated with synaptic functioning in post-mortem brain tissue of SZ versus control subjects (Jaffe et al., 2018). Moreover, structural and functional alterations of neuronal circuits have been observed (Wu et al., 2012), such as receptor modifications, dendritic spine adjustments, and postsynaptic density size reduction (Konopaske et al., 2014; McCollum et al., 2015). It was suggested that *the synaptic changes and functional dysconnectivity observed in SZ patients are linked to E/I imbalance at the level of cortical microcircuitry*, which influences cortical synchrony at the macroscale level (Stephan et al., 2006; Yizhar et al., 2011). Synchronized neural oscillations, in turn, influence cortical network plasticity (Huerta and Lisman, 1993; Singer and Gray, 1995) and are crucial for cognitive functions. Combined alterations in synaptic transmission, long-term synaptic plasticity, and synchronous oscillations seem to underpin CIAS (Uhlhaas and Singer, 2010). Altered presynaptic Ca^2+^ signaling was proposed to dysregulate LTP and to play a role in CIAS (Nanou and Catterall, 2018; Pereda et al., 2019; Wu et al., 2022).

Several lines of evidence support the role of altered synaptic plasticity in CIAS. LTP and LTD proved to be affected both in clinical and preclinical subjects (Wu et al., 2022) and were significantly associated with the course of the disease (Hasan et al., 2011). TMS and transcranial direct current stimulations (tDCS) revealed impaired LTP-like plasticity due to dysfunctional NMDAR and GABAR (Hasan et al., 2011; Hamilton et al., 2020). In rodents with psychotic symptoms induced by MK-801, LTP following high-frequency stimulation is disrupted (Frankiewicz et al., 1996; Obi-Nagata et al., 2019). This effect may be related to NMDAR hypofunction in GABAergic neurons resulting in E/I imbalance and impaired synaptic plasticity, and results in a range of cognitive deficits, including attention, memory, and learning, while also contributing to hallucinations and delusions (Nakazawa and Sapkota, 2020).

Beyond functional evidence, genetic markers of synaptic plasticity showed alterations in neural cell adhesion molecule-1, Neurotropin-3, and Matrix-metalloproteinase-9 in CIAS (Keshri and Nandeesha, 2023). Large-scale gene expression studies evidenced a reduction in the presynaptic protein synaptophysin in hippocampus, frontal cortex, and cingulate cortex (Osimo et al., 2019). PET imaging revealed that synaptic vesicle glycoprotein 2A, widely expressed in presynaptic terminals and synaptic vesicles, was reduced in different brain regions in SZ, and was associated with either positive or cognitive symptoms (Onwordi et al., 2021; Radhakrishnan et al., 2021). Post-mortem and GWAS findings were confirmed by transcriptomic and proteomic data obtained from patient-derived induced pluripotent stem cells (Santarriaga et al., 2023).

In summary, functional and genetic findings obtained using multiple techniques support alteration in synaptic transmission and plasticity in SZ (Abashkin et al., 2021; Nascimento et al., 2022) opening the question on how and when these changes happen. During neurodevelopment, genetic and environmental factors make synapses vulnerable to stress-triggered glia-mediated elimination, disrupting neuron function and worsening symptoms like psychosis (Howes et al., 2023). This can be especially relevant to SZ, typically emerging during adolescence or adulthood. Since *neuronal plasticity is a critical factor implicated in CIAS*, it may be targeted to improve synaptic plasticity and cognitive performance in SZ (Mould et al., 2021). Again, the cerebellum presents major plastic mechanisms that might be relevant for CIAS and will be considered below (Section 3.2).

### 2.3 Abnormal connectivity

Dysconnectivity refers to disruption in communication and coordination between brain regions. *The disconnection hypothesis* (Friston et al., 2016) posits that a failure of functional integration occurs in the SZ brain. Accordingly, the alterations of structural and functional connectivity within and between brain regions is a core hypothesis of CIAS (Repovs et al., 2011; Frangou, 2014; Canu et al., 2015; Adhikari et al., 2019; Uyy et al., 2020). The analysis of resting state (RS) networks implicated in cognitive control, task set maintenance, attention, and error processing (Fair et al., 2009) suggested that not only the cerebral cortex, primarily the *prefrontal and limbic cortex*, but also subcortical hubs, including *cerebellum, thalamus, and basal ganglia*, play an important role in the pathogenesis of SZ (Andreasen et al., 1996; Andreasen and Pierson, 2008; van den Heuvel and Fornito, 2014; Rolls et al., 2020). An fMRI study demonstrated that connectivity alterations are circuit-specific, with *prefrontal-limbic hypoconnectivity and primary-sensorimotor hyperconnectivity* extending consistently across subcortical nuclei (Avram et al., 2018). However, variations are reported depending on the stage of development of the disease (Kochunov et al., 2017; Anhøj et al., 2018; Sharma et al., 2018; Chen et al., 2019; Hu et al., 2023).

Dysconnectivity could explain distinct symptom dimensions (Avram et al., 2018) and correlated with social cognition, reasoning/problem-solving, and working memory capabilities (Zarghami et al., 2023). Dysconnectivity has been also related to specific neurotransmitter systems. A PET study suggested that aberrant striatal dopamine and cortico-thalamic connectivity are physiologically related within dopamine-modulated cortico-basal ganglia-thalamic circuits in SZ. Moreover, the disconnection between medial PFC and the dorsal hippocampus was related to CIAS in a rodent model of NMDAR hypofunction and was partially rescued by Risperidone (one of the most prescribed atypical antipsychotic drugs primarily targeting D2R and 5-HT2AR receptors and known to improve executive function, attention, learning, and memory) (Delgado-Sallent et al., 2023).

In conclusion, brain dysconnectivity plays a vital role in the pathophysiology of SZ, affecting multiple brain networks and contributing to cognitive impairment. A primary role is apparent for brain circuits involving associative areas, including the prefrontal, temporal, and limbic cortex. Nonetheless, findings across studies show a range of changes, including reduced connectivity in some networks (thalamic-frontal, left frontoparietal, lateral and medial visual, sensorimotor, DMN, and auditory) and increased connectivity in others (right central executive, right ventral attention, subcortical nuclei networks). Moreover, dysconnectivity correlates with distinct symptom dimensions and is associated with specific neurotransmitter systems. The involvement of cerebellum, among the subcortical regions, has also emerged and will be considered in Section 3.3.

### 2.4 Abnormal neurodevelopment

The neurodevelopmental deficit is a fundamental concept in the pathophysiology of SZ and provides an ontogenetic framework for modifications in neurotransmitters, connectivity, and synaptic plasticity. Weinberger hypothesized that SZ symptoms, despite appearing in early adulthood, stem from environmental and genetic factors causing abnormal prenatal brain development (Weinberger, 1987). The onset of the illness occurs during a vulnerable period in adolescence when neural alterations may be activated (Fatemi and Folsom, 2009). Synaptic formation and maintenance occur during the second and third trimester of pregnancy, then synaptic connectivity develops during childhood. These ontogenetic changes are crucial for learning, memory, and brain functioning. Alterations in synaptic development can lead to SZ and ASD (Hall and Bray, 2022). The onset of SZ in adolescence can be related to the “plasticity switch” secondary to the peripubertal brain maturational changes, caused by modifications in the glutamatergic system. The loss of plasticity could result in social and non-social cognitive deficits (Keshavan and Hogarty, 1999). Synaptic pruning with excessive elimination of synapses and loss of synaptic plasticity alters microconnectivity and can lead to the emergence of symptoms in the predisposed brain. Another possible mechanism is myelination of the heteromodal association cortex that proceeds postnatally. During adolescence (Peters et al., 2012), aberrant myelination and oligodendrocyte number may contribute to connectivity dysfunction in SZ.

The neurodevelopmental hypothesis explains why prodromal symptoms of SZ start during adolescence. Likewise, individuals who will later develop SZ may exhibit non-specific indications of mild brain dysfunction before the onset of the disease, which can be observed as subtle motor abnormalities or cognitive impairments (Cuesta et al., 2018). Cognitive decline continues from the first episode through the chronic stage (Bonner-Jackson et al., 2010; Sheffield et al., 2018). Although cognitive deterioration is common in all stages of SZ, deficiencies in executive functions (e.g., learning, processing speed, organization) are more common in the chronic stage (Stone and Seidman, 2016). In clinical high-risk SZ adolescents, progressive grey matter reduction in the right superior frontal, middle frontal, and medial orbitofrontal cortical regions, as well as a greater rate of expansion of the third ventricle, were observed (Cannon et al., 2015). This was replicated in a subsequent meta-analysis (Ding et al., 2019). In addition, white matter abnormalities, pointing to a neurodevelopmental pathology, were observed (Seitz-Holland et al., 2023).

Genetic alterations are thought to lay at the basis of the aberrant neurodevelopmental processes in SZ and are evaluated using the *polygenic risk score* and its correlations with clinical and anatomo-functional parameters (Cattarinussi et al., 2022; Fernandez-Cabello et al., 2022).

A factor that could operate during pregnancy is maternal infection with the consequent immune activation that impairs dendritic spine development and synaptic plasticity (Pekala et al., 2021). Reduced synaptic plasticity along with reduced dendritic spines, decreased expression of synaptic genes, and abnormal synaptic neurotransmission have been reported in SZ (Berdenis van Berlekom et al., 2020). Indeed, reduced dendritic spine density (together with reduced parvalbumin interneurons) is a characteristic histopathological feature of SZ. *The loss of microconnectivity* can cause aberrant myelination, impaired connectivity, and cognitive deficits (Valdés-Tovar et al., 2022; Wu et al., 2022). The interaction between genetic and environmental insults linked to the neurodevelopmental model in SZ has been recently reviewed in detail (Schmitt et al., 2023). Prenatal and perinatal complications, childhood trauma, and maternal immune activation interact with genetic susceptibility to shape neurodevelopmental trajectories and increase the risk of developing SZ (Schmitt et al., 2023).

Relevant to this review, a recent Polygene score analysis evidenced the role of the cerebellum and its connectivity in neurodevelopmental psychiatric disease, suggesting that the genetic patterning for child psychopathology is distinct from that for adults, and implicates fetal cerebellar development (Hughes et al., 2023). A better evaluation of *cerebellar neurodevelopmental abnormalities* is warranted, given the identification of over 1,000 genes in the cerebellum related to neurodevelopmental disorders (Sepp et al., 2024).

## 3 The potential role of cerebellum in CIAS

**Figure 3:**
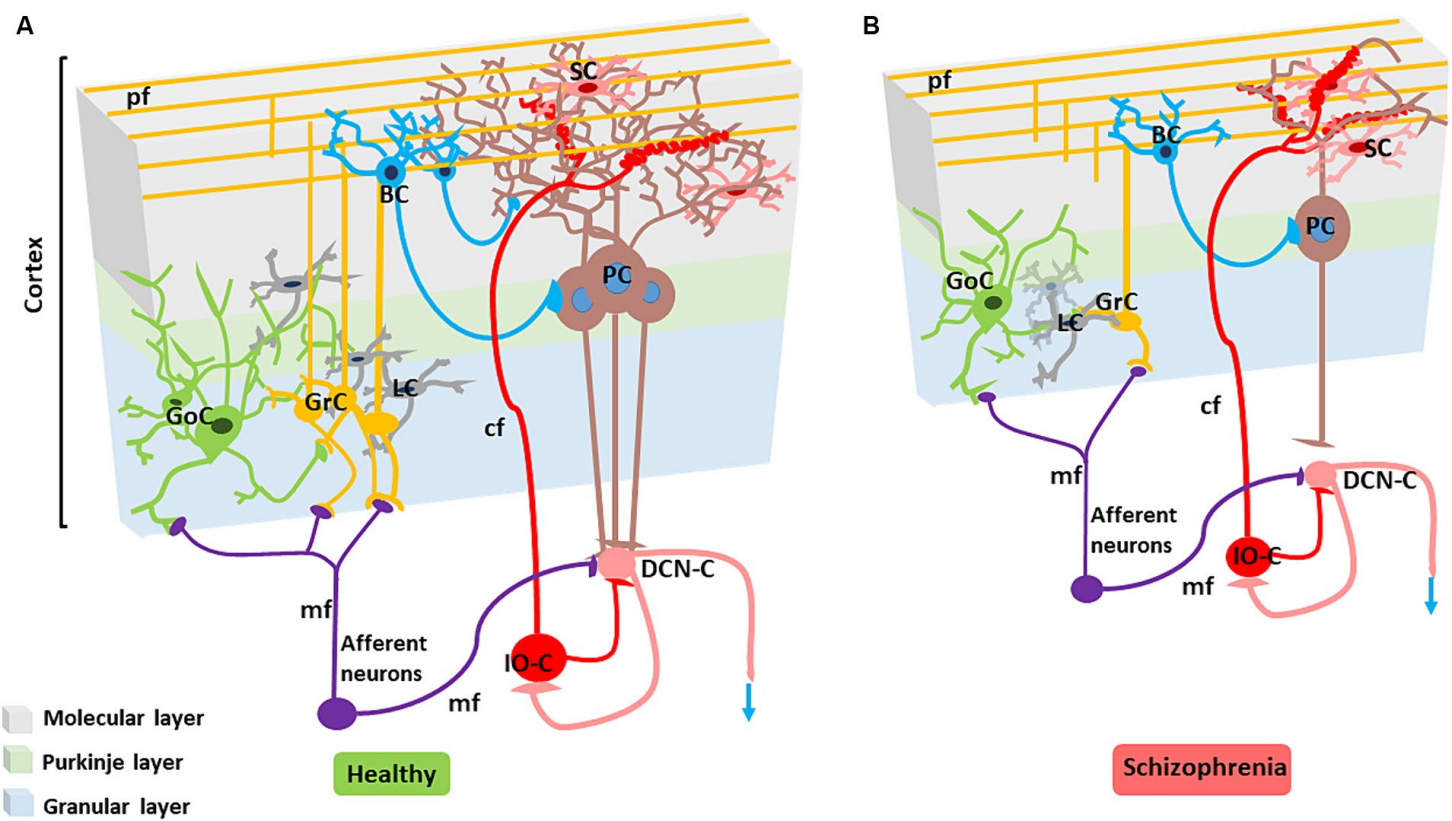
Alterations in the SZ cerebellar microcircuit. **(A)** Healthy cerebellar microcircuit: The simplified circuit scheme includes cortical (grey area) and subcortical structures. Afferent fibers activate the cerebellar cortex as well as DCN cells (DCN-C) and IO cells (IO-C), then the DCN emits the output and inhibits the IO. The cerebellar cortex is therefore a large side loop controlling DCN activity. The cerebellar cortex contains various types of neurons, primarily granule cells (GrC), Golgi cells (GoC), Purkinje cells (PCs), stellate cells (SC) and basket cells (BC). Other neurons, including Lugaro cells, unipolar brush cells, candelabrum cells, and globular cells are not shown. The two primary inputs are mossy fibers (mf), originating from various brainstem and spinal cord nuclei, and climbing fibers (*cf*) originating from the IO. Signals conveyed through the mossy fibers diverge, activating the DCN and the granular layer (containing GrC and GoC). The ascending axon of the GrC bifurcates in the molecular layer (containing PC, SC, and BC), forming the parallel fibers (pf). The cerebellar cortical circuit consists in a forward excitatory neuronal chain forming multiple inhibitory loops: mossy fibers excite GrCs, which subsequently activate all the other cortical elements. In the granular layer, inhibition is provided by GoC, and in the molecular layer by SC and BC. Finally, PCs inhibit the DCN. The IO, which is also activated by brainstem and spinal cord projections, controls PC activity through a single powerful synapse. Consequently, the entire system can be seen as a complex mechanism regulating the DCN output. Re-drawn from D'Angelo et al. (2016). **(B)** Cerebellar microcircuit in schizophrenia condition: The figure illustrates the main alterations reported in the SZ cerebellar microcircuit. The grey matter and white matter thickness are decreased. Microscopic alterations include reduced density of the main neuronal populations (PCs, GrCs, inhibitory interneurons), reduced PC dendritic branching, altered synaptic vesicular transport (not shown), increased connectivity at climbing fiber/PC synapses, disconnection between PC and neuronal populations in the DCN.

Following the identification of the *CCAS* (Schmahmann and Sherman, 1998; Schmahmann, 2016; Argyropoulos et al., 2020) and the core hypothesis on cognitive dysmetria (Andreasen et al., 1998; Andreasen and Pierson, 2008), new evidence calls for updating the role of cerebellum in CIAS (see Figure 3 and Sections 1.1, 1.2, 1.3 in Supplementary material for details on cerebellar anatomy and physiology):

1. *Cognitive domain*: The multiscale analysis of circuit operations supports the cerebellar involvement in the main cognitive domains of CIAS, *attention, learning, decision-making* (D'Angelo and Casali, 2012), akin to the involvement of cerebellum in cognitive, emotional, and behavioral control (Ciapponi et al., 2023; Supplementary Figure S1).
2. *Processing speed*: The cerebellum contributes substantially to mechanisms of CIAS, like *processing speed*, by allowing mental processing to move from controlled to automatic mode (Wong et al., 2021).
3. *Connectivity*: The cerebellum shows tight bidirectional connectivity with associative areas involved in CIAS, especially the PFC (Palesi et al., 2015, 2017, 2018).
4. *Microcircuit level*: Cerebellar functioning relies on a delicate regulation of the internal E/I balance, which appears altered in CIAS (D'Angelo and De Zeeuw, 2009; Mapelli et al., 2014; Nieus et al., 2014; Perez-Garcia, 2015; De Schepper et al., 2022).
5. *Whole brain level*: The cerebellum controls the functioning and rhythms of the cerebral cortex (Popova and Naumenko, 2013; Margarint et al., 2020), which show relevant alterations in CIAS.
6. *Neuromodulation systems*: The cerebellum is emerging as part of complex regulatory systems that subtend CIAS and are based on dopamine (Ikai et al., 1994; Carta et al., 2019; D'Angelo, 2019; Cutando et al., 2022; Kimura et al., 2023), Ach (Jaarsma et al., 1997; Zhang et al., 2016; Okkels et al., 2023; Zhao et al., 2023), and 5HT (Oostland and van Hooft, 2013; Saitow et al., 2013). Moreover, the cerebellum hosts among the most important NMDA receptor-dependent neurotransmission and plasticity mechanisms in the brain, addressing the glutamatergic hypothesis of CIAS (Hansel et al., 2001; Contestabile, 2002; Bouvier et al., 2016; Mapelli L. et al., 2022).

### 3.1 Cerebellar structural and functional abnormalities and CIAS

Cerebellar Crus I- II, VIIB, and, to a lesser extent, VIIIA and VI, are linked to executive functions and cognitive control and deficits in tasks requiring flexibility, inhibition, and goal-directed behavior can emerge from damage to these areas (Schmahmann and Sherman, 1998; Argyropoulos et al., 2020). Posterior cerebellar lesions are associated with deficits in executive functions that resemble those observed in prefrontal lesions, including impairment in planning, verbal fluency, working memory, problem-solving, and multi-task performance and organization (Schmahmann and Sherman, 1998, Argyropoulos et al., 2020), while lesions in vermis and paravermis regions are associated with behavior alteration and mood disturbance. Likewise, cerebellar degeneration can cause impairments of executive function, working memory, and perceptual processing (Kansal et al., 2017). Verbal and phonemic fluency, working memory, cognitive flexibility, immediate and delayed recall, verbal learning, and visuomotor coordination were variably associated with lobule VI, Crus I- II, VII B, and/or IX, whereas immediate, and delayed recall show associations with the anterior lobe.

Cerebellar abnormalities in psychosis are tied not only to a specific diagnosis or illness stage but also to developmental factors and premorbid cognitive disturbances (Moussa-Tooks et al., 2022). Cerebellar dysfunction has been repeatedly correlated with CIAS (Andreasen and Pierson, 2008; Dean and Porrill 2014; Ding et al., 2019, Kim et al., 2021). Neuroimaging studies reported smaller cerebellar volume, altered intra-cerebellar and cerebellar-cerebral RS functional connectivity, and reduced cerebellar activation during cognitive tasks (Zhuo et al., 2018; He et al., 2019; Kim et al., 2021; Lundin et al., 2021; Moussa-Tooks et al., 2022). On a microscopic scale, neuropathological changes include lower Purkinjie Cell (PC) density and reduced distal and terminal dendritic branches (Mavroudis et al., 2017). PCs and granular cells (GrCs) are key in maintaining the E/I balance but, in SZ, deficits in the development of PCs and GrCs (and possibly also in other cell types) could alter the E/I balance required for cerebellar network functioning (Perez-Garcia, 2015; Figure 3).

First-episode SZ patients exhibit reduced cerebellar grey matter and altered functional activation prominently in lobules IV, V, VII, and VIII, and in Crus I-II (Ding et al., 2019; Kaufmann et al., 2019; Li X. et al., 2022; Li Y. et al., 2022). Moreover, mean age and illness duration were negatively associated with the reduction in the left Crus II (Li X. et al., 2022). A correlation study between cerebellar anatomy and functional activation with cognitive scores revealed that anatomical characteristics predicted both cognitive abilities and psychopathology (Moberget et al., 2019) [but see Guo et al. (2018) and Moussa-Tooks et al. (2022)]. A recent preclinical study reported an increase in climbing fiber/Purkinje cell synaptic connectivity following neonatal subchronic administration of Phencyclidine (PCP), a drug of abuse with psychomimetic effects leading to long-term behavioral changes related to SZ in rodents (Veleanu et al., 2022). In a post-mortem study, cerebellar cortex abnormalities correlated with the altered expression of 23 genes involved in cerebellar presynaptic vesicular transport, Golgi function, and GABAergic neurotransmission (Mudge et al., 2008).

In SZ patients, cerebellar cortex volume is significantly decreased, with the most pronounced effects observed in regions functionally connected with frontoparietal cortices. This has been consistently reported as one of the most prominent structural alterations, alongside other changes such as reductions in hippocampus volume and frontotemporal cortical thickness. Positive correlations emerged between cerebellar volume and cerebral cortical thickness in frontotemporal regions, suggesting common underlying disease processes jointly affecting the cerebellum and the cerebrum. Interestingly, cerebellar volume reduction in SZ was highly consistent across the age span 16–66 years and was present already in the youngest patients, which is more in line with neurodevelopmental than neurodegenerative etiology (Moberget et al., 2018, 2019).

Bègue and co-workers used canonical correlation analyses to link cerebellar grey matter volume to cognitive functioning. They proposed two maps: one associated with cognitive flexibility, processing speed and working memory (Crus II and Lobule X) and the other with working memory (Crus I and Lobule VI), both linked also to working memory (Bègue et al., 2023). While cerebellar volume reduction is the most common finding, an increase in right cerebellum and lingual gyrus grey matter volume was also associated with formal thought disorders in SZ patients (Maderthaner et al., 2023).

**Figure 4:**
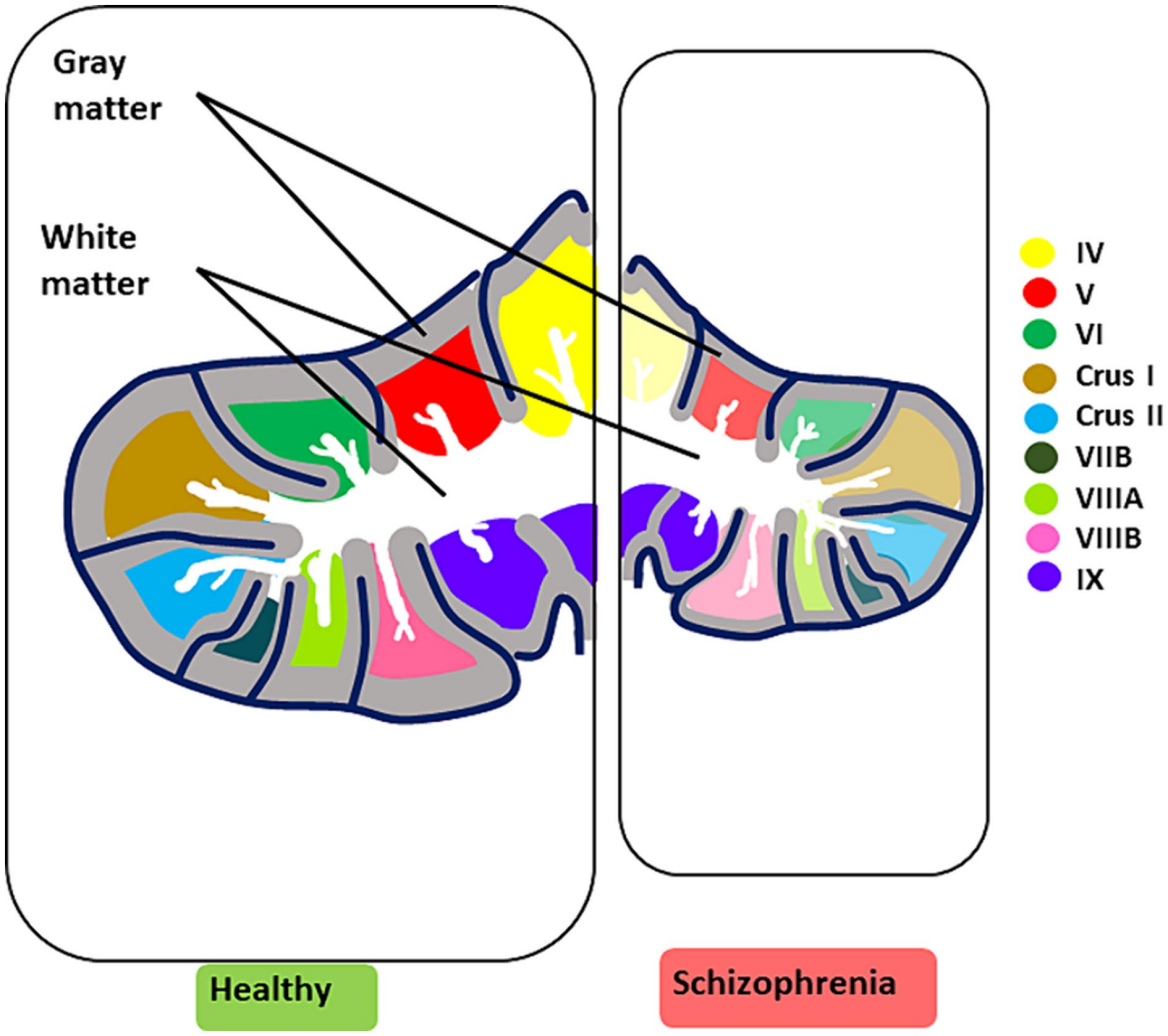
Key structural and functional alterations in the SZ brain. The illustration summarizes the most prominent cerebellar structural and functional alterations observed in CIAS. Structural alterations are characterized by an overall reduction in volume, accompanied by a decrease in grey matter and white matter thickness (for microscopic alterations see Figure 3). Reduced functional activation in CIAS is observed in cerebellar lobules IV, V, VI, Crus I and II, VIIB, VIIIA, and VIIB. Each lobule is color-coded for clarity. In schizophrenia (right panel), the affected lobules are colored in pale shades.

Finally, sexual dimorphisms have been considered in normative cerebellar developmental trajectories, challenging the belief that males have inherently larger cerebellar volumes, possibly due to differences in hormonal fluctuations and environmental experiences (Sefik et al., 2022). In clinical high-risk SZ females, smaller cerebellar cortex sizes correlated with more severe disorganization symptoms, especially in negative-related domains, while SZ males under 20 showed reduced white matter volume (Sefik et al., 2022) (see Figure 4 for cerebellar structural/functional alterations and Figure 3 for microcircuit abnormalities).

### 3.2 Cerebellar neurotransmitters alteration in CIAS

**Figure 5:**
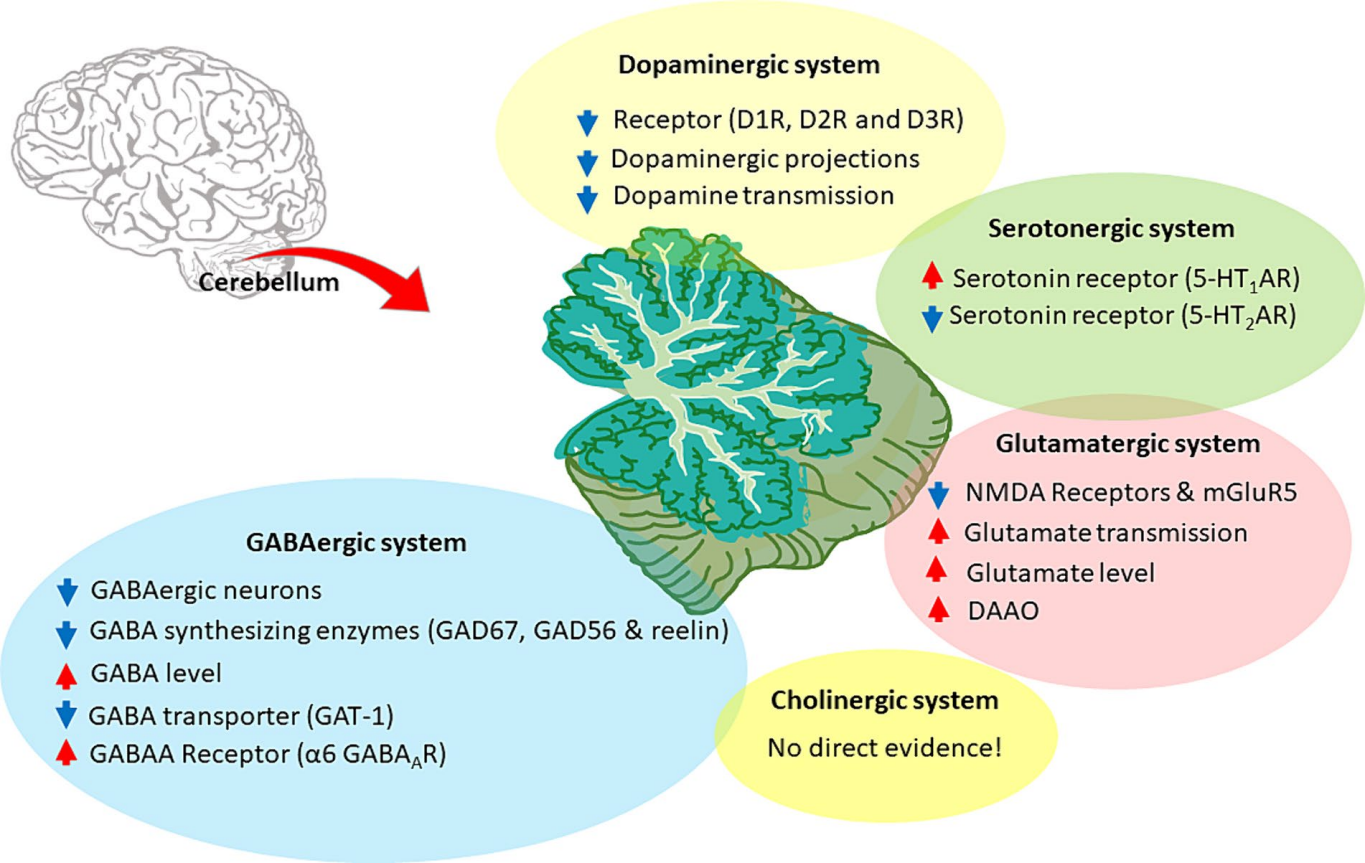
Neurotransmitter alterations in the SZ cerebellum. The figure illustrates the cerebellar neurotransmitter systems and their hypothetical relationship with SZ. Hyperfunctioning and hypofunctioning are identified with red and blue arrows, respectively. Cerebellar dopaminergic system: dysregulation of dopamine receptors (DR), particularly D1R and D2R, would decrease dopaminergic transmission. Alterations also involve aberrant dopaminergic projections and overall downregulation of dopamine signaling. Cerebellar glutamatergic system: the hypothesis focuses on the hypofunction and downregulation of glutamate receptors, particularly NMDAR and mGluR5. It further includes the acceleration of glutamate transmission, release, and related enzymes, suggesting an altered glutamatergic system in the cerebellum. Cerebellar GABAergic system: the hypothesis involves various aspects, such as decreased GABAergic projections, aberrant levels of synthetic enzymes (like GAD67 and GAD56), and elevated GABA concentration. It also highlights specific GABA receptors, particularly α6 GABAAR, along with lower expression of GABA transporters, such as GAT-1. Cerebellar cholinergic system: there is no direct evidence for the involvement of the cerebellar cholinergic system in CIAS. Cerebellar Serotonergic system: some alterations were found in SZ, such as 5-HT_1_AR upregulation and 5-HT_2_AR downregulation.

Like the forebrain, the cerebellum is regulated through a complex system of neurotransmitters (Ottersen, 1993). Fast excitatory synaptic transmission is mediated by glutamate, and inhibitory synaptic transmission is mostly mediated by GABA (although there are also glycinergic synapses). Glutamate is released from mossy fibers (mfs) onto GrCs (D'Angelo et al., 1990) which, in turn, release glutamate onto PCs. GABA regulates the overall excitability of the cerebellar circuit. GABAergic neurons (Purkinje cells, Golgi cells, stellate cells, and basket cells) provide inhibitory signals that fine-tune and modulate the output from the cerebellum to other parts of the brain (Mapelli et al., 2014; Nieus et al., 2014). In addition to GABA and glutamate, several neuromodulators, such as dopamine, serotonin, noradrenaline, and acetylcholine, play important roles in modulating cerebellar function (Zhang et al., 2016). For example, while the cerebellum is not classically considered an elective dopaminergic region, recent studies showed that it has an important involvement in dopaminergic control and plays a role in dopamine deficit-related neurological and psychiatric disease (Flace et al., 2021). Serotonin is known to modulate GABAergic and glutamatergic signaling in the adult cerebellum, where it can adjust PCs and Lugaro cell firing rate (Dieudonné and Dumoulin, 2000; Fleming and Hull, 2019). Ach can enhance glutamatergic neurotransmission and plasticity in the cerebellar glomeruli (Prestori et al., 2013). Eventually, these neuromodulators can influence cerebellar functioning impacting motor learning and control as well as cognitive processing (Ankri et al., 2015; Mapelli et al., 2015; Gao et al., 2016; Flace et al., 2021). Since dysfunction and imbalance in neurotransmitter systems lead to various neurological disorders including CIAS, addressing the intricate interplay of cerebellar neurotransmitters could provide insight into the underlying causes of these disorders and allow for the development of targeted treatments to restore cognitive decline in SZ. The evidence supporting the impact of cerebellar neurotransmitters in CIAS is presented below and summarized in Figure 5.

#### 3.2.1 The cerebellar dopaminergic system

As seen above, dopamine in forebrain circuits is crucial for cognitive functioning and is significantly implicated in SZ pathophysiology (Davis et al., 1991; Kesby et al., 2018) (Section 2.1). A fact that has not been sufficiently recognized before is that dopamine is also present at high concentrations in the cerebellum. In rodents, the deep cerebellar nuclei (DCN) show higher dopamine concentrations than hippocampus and cerebellar cortex, and similar to frontal cortex (Versteeg et al., 1976). Several clinical and preclinical studies showed a crucial involvement of cerebellar dopaminergic mechanisms in CIAS (Demirtas-Tatlidede et al., 2010; Rogers et al., 2013; Parker et al., 2014).

Recently, the cerebellar dopaminergic system has been characterized. Receptor subunits D1R-D5R have been reported in various lobules of the cerebellar cortex, mostly in PCs, where they impact synaptic and cellular plasticity (Cutando et al., 2022). Dopaminergic projections to the cerebellar cortex and nuclei originate mainly from VTA (Ikai et al., 1994) and in part from locus coeruleus (Canton-Josh et al., 2022) that also gives rise to the major dopaminergic midbrain and cerebral pathways (Panagopoulos and Matsokis, 1994). Moreover, PCs produce dopamine (Li et al., 2023). Finally, the cerebellum output through the DCN regulates the VTA (Carta et al., 2019) and the substantia nigra (Washburn et al., 2024). Therefore, *the cerebellum has a triple relationship with the dopaminergic system: it produces dopamine, it receives dopaminergic innervation, and it regulates the dopaminergic systems of the brain stem and basal ganglia.*

PCs synthesize and release dopamine in an activity-dependent manner modifying local microcircuit functioning (Li et al., 2023). Dopamine binds to D1Rs in Bergman glial cells causing membrane depolarization and activating a Ca^2+^ signaling cascade leading to AMPA receptor GluA1 subunits membrane insertion and glutamate release. This, in turn, enhances interneuron activity reducing PC excitation by parallel fibers and climbing fibers (cfs) and altering the PCs firing frequency and pattern, eventually impacting locomotor and social behavior. These findings indicate that the cerebellar dopaminergic system has a critical pathophysiological role in disorders associated with motor and social dysfunction (Li et al., 2023). Axons coming from the locus coeruleus may regulate cerebellar cortex activity by co-releasing dopamine onto D1R-positive unipolar brush cells. PCs, then directly inhibit the same unipolar brush cells, forming a dopamine-sensitive recurrent circuit (Canton-Josh et al., 2022).

The cerebellum modulates VTA dopamine release via direct projections impacting the expectation/reward mechanism (Carta et al., 2019; Holloway et al., 2019). This pathway extends the role of cerebellum in error detection to the discrepancy between motivation and expectation of reward allowing a cerebellar control on emotional and social behavior (D'Angelo, 2019).

As seen above, hypoactivity in the mesocortical pathway is associated with negative symptoms and CIAS (Toda and Abi-Dargham, 2007; Treadway and Zald, 2011). Interestingly, reduced functionality in cerebellar circuits alters dopaminergic activity in the medial PFC (Rogers et al., 2011), suggesting that a third, cerebellum-related, control system impacts dopaminergic functions in SZ. Indeed, electrical stimulation of the Purkinje layer and DN evokes a long-lasting increase in dopamine release in PFC. Thus, a disconnection between the PCs and neuronal populations of the DN could alter dopaminergic signaling in PFC and impact SZ symptoms (Mittleman et al., 2008) and CIAS. Reduced interaction between the cerebellum and the basal ganglia-dopamine network might be involved in regulating the motivation domain (Yoshida et al., 2022).

Indirect evidence supports this hypothesis. (1) In rat cerebellum, the atypical antipsychotic blonaserin and the anxiolytic buspirone engage extensively in D3R regulation and their action is associated with cognitive impairment (Baba et al., 2015; Di Ciano et al., 2017). (2) A reduced cerebellar expression of SP transcription factors and D2Rs was related to negative symptoms observed in SZ (Pinacho et al., 2013). (3) In genomic DNA isolated from the cerebellum, the atypical antipsychotic agent olanzapine increased methylation of genes related to the dopaminergic system, such as *D3R*, DOPA decarboxylase, and *VMAT2* (*SCL18A2/VMAT2*) (Melka et al., 2013). (4) Alteration in D2R levels in PCs of male mice during adulthood alters sociability and preference for social novelty without affecting motor functions (Cutando et al., 2022). (5) Aberrant dopamine neurotransmission in SZ influences the cerebellar vermis affecting time processing and directly addressing the cognitive dysmetria hypothesis (Yeganeh-Doost et al., 2011). While more research is warranted, a causal link is beginning to emerge between cerebellar dopamine and CIAS.

#### 3.2.2 The cerebellar cholinergic system

Cholinergic signaling in the cerebral cortex and basal forebrain is strongly related to learning and memory (Lecrux et al., 2017) (Section 2.1) but its role in the cerebellum is less explored. Nevertheless, there is growing evidence indicating that cholinergic projections from the brainstem may influence cerebellar function and play a modulatory role in cognitive processing. Cholinergic projections form the third afferent system of the cerebellum, following cfs and mfs (de Lacalle et al., 1993), and seem to play a modulatory role by biasing neuronal excitability and synaptic responses, eventually influencing the cerebellar output and behavioral responses (Zhang et al., 2016).

Nearly half of cholinergic neurons in the brainstem project to cerebellum (Zhao et al., 2023). The vermis also harbors a substantial population of cholinergic neurons, and a dysfunction in this region may potentially contribute to cognitive deficits (Huang et al., 2007), as observed in Parkinson’s disease patients (Maiti et al., 2020). Both nAChRs and mAChRs are expressed in the cerebellum and are activated by Ach released from cholinergic fibers.

In rodents, mAchRs are present in all cerebellar lobules with differential expression across layers. Expression is higher in the PC layer of lobules I-V, Crus I-II, in the GC layer of lobules VI-VII, and in the molecular layer of all other lobules. Cholinergic fibers emerge from the inferior peduncle and spread across the cerebellar cortex as mfs, glomerular rosettes, and thin varicose fibers (Jaarsma et al., 1997). The cerebellum shows choline acetyltransferase (ChAT) (Jaarsma et al., 1997) and acetylcholine esterase (Okkels et al., 2023) activity, and [^18^F] FEOBV PET imaging have revealed Ach uptake in the cerebellum *in vivo*, most markedly in the vermis and flocculonodular lobe (Okkels et al., 2023).

The α7-nACh receptor subunit controls LTD/LTP balance at mf-GrC synapses, which, consequently, facilitates neural adaptation (Prestori et al., 2013). Similarly, applying nicotine during an air-puff stimulation task influences GrCs activity (Xu et al., 2019). Cerebellar nAChRs can also regulate GABA release from interneurons in a subtype-specific manner and affect cognitive functions (Turner et al., 2011).

Alterations of the cerebellar cholinergic system were documented in various mental disorders and associated with cognitive decline. For instance, in a rat model of Japanese encephalitis characterized by marked damage in cognitive functions, transient spatial learning and memory deficits were due to reduced cholinergic activities in various brain regions including the cerebellum (Chauhan et al., 2016). The reduction involved most cholinergic markers, including total muscarinic receptor bindings and M2 receptor, CHRM2 mRNA level, and ChAT expression (Chauhan et al., 2016).

Ach plays a crucial role in the cerebellar interpositus nucleus during execution and coordination of voluntary movements, through activation of muscarinic receptors. Moreover, the cholinergic system is relevant for reward-related behavior (Pickford et al., 2023). Bilateral cerebellar infusion of scopolamine (mAChR antagonist), or Mecamylamine (mAChR antagonist) differentially impaired motor performance (Pickford et al., 2023). Disruption in cholinergic neurotransmission has been associated with executive dysfunction in animals and humans affected by SZ (Section 2.1). While there is currently *no direct evidence evaluating the role of cerebellar cholinergic signaling in CIAS*, alterations in the cholinergic system might impact CIAS. For instance, individuals with SZ often experience cognitive impairments in working memory, attention, and executive functions, all of which have been associated with cerebellar activity and cerebellar cholinergic signaling. These observations are indirect and further research is required to elucidate the relationship between cerebellar cholinergic dysfunction and CIAS.

#### 3.2.3 The cerebellar serotonergic system

Serotonergic signaling regulates mood, cognition, and various physiological processes, and its alteration is implicated in SZ pathophysiology (Section 2.1). Although serotonin is commonly associated with PFC and limbic system, it is also present in the cerebellum (Oostland and van Hooft, 2013), and serotonergic fibers represent one of the primary input pathways. Cerebellar 5-HT modulates glutamatergic and GABAergic synaptic transmission, regulates signal flow in PCs, facilitates firing, and regulates synaptic transmission and long-term synaptic plasticity in DCN neurons (Saitow et al., 2013). In the cerebellar cortex and DCN, different subtypes of serotonergic receptors (5-HT_1_B, 5-HT_2_B, 5-HT_2_A, 5-HT_3_, and 5-HT_5_A) have been identified (Duxon et al., 1997; Oostland and van Hooft, 2013).

The serotonergic system controls cerebellar development and is implicated in neurodevelopmental diseases (Oostland and van Hooft, 2013). Initially, 5-HT regulates dendritic growth and synaptic plasticity. In the first postnatal week, activation of 5-HT₁R expressed by GrCs and PCs stimulates dendritic growth and synapse formation (Oostland et al., 2014). Then, activation of 5-HT₃ Rs in GrCs limits dendritic growth of PCs by modulating pf-PC plasticity and *cf* competition for PC dendrites. Finally, activation of 5-HT₂R in GrCs and PCs during late postnatal development and in the mature cerebellum stabilizes synaptic activity (Oostland and van Hooft, 2013; Oostland et al., 2014). The Lugaro cells are also specifically targeted by serotonergic inputs that can increase their firing thereby inhibiting Golgi cells (GoC) (Dieudonné and Dumoulin, 2000).

Under physiologic conditions, cerebellar 5-HT_1_ARs decline during the neonatal stage and disappear by early childhood. In contrast, in SZ, cerebellar 5-HT_1_ARs persist in adulthood, specifically in the vermis, in relation to abnormal serotonergic innervation (Slater et al., 1998). These results support the notion that SZ has a neurodevelopmental component and that cerebellar 5-HTRs expression goes wrong during ontogenesis. This aspect is intriguing since 5-HT_1_AR is strongly associated with disturbed mood and emotion (Popova and Naumenko, 2013). Upregulation of cerebellar 5-HT_1_AR in SZ has been confirmed by *in vivo* PET imaging (Tauscher et al., 2002) while immunolabeling revealed that cerebellar 5-HT_2_AR is reduced in SZ subjects (Eastwood et al., 2001).

The reported alterations in 5-HTR expression in the cerebellar cortex and nuclei contribute to the serotonergic hypothesis of SZ, although a direct demonstration is still lacking. Please note that altered cerebellar serotonergic signaling is potentially associated with ASD, where a broad distribution of 5-HT_5_A mRNA has been revealed in all cerebellar regions (Marazziti, 2002).

#### 3.2.4 The cerebellar glutamatergic system

Alterations of the glutamatergic system in SZ (see Section 2.1) have been reported not just in the basal ganglia, temporal lobe, and thalamus (Merritt et al., 2023) but also in the cerebellum. The cerebellum contains the highest concentration of NMDA receptors in the brain along with a rich variety of receptor subtypes and receptor-dependent mechanisms. A recent preclinical study suggested the crucial role of cerebellar glutamatergic neurotransmission during brain development in motor and social behavior (van der Heijden et al., 2023).

Cerebellar NMDARs are crucial for neuronal survival (Contestabile, 2002) and circuit development and functioning (Rabacchi et al., 1992; Bidoret et al., 2009). Repeated ketamine administration causes neurodegeneration in the cerebellum and memory loss in rats (Onaolapo et al., 2019). PCP administration during the neonatal stage impacts development of the olivocerebellar circuit. In the PCP model, the mRNA levels of two GoC selective NMDAR subunits, NR2B and NR2D, decreased (Bullock et al., 2009). In humans, in the first episode of psychosis, individuals displayed elevated levels of glutamate both in the associative striatum and cerebellum (de la Fuente-Sandoval et al., 2013). All NMDAR subunits are expressed in the cerebellum, with significant expression of GluN2C and GluN2D (Thompson et al., 2000), and might be altered in SZ (Schmitt et al., 2010).

Alterations in cerebellar NMDAR expression and activity were implicated in cerebellar circuit dysconnectivity and strongly correlated with CIAS (Yeganeh-Doost et al., 2011). The expression of the NR2C subunit in mature mf-GrC synapses (Mullasseril et al., 2010) is regulated by NRG1 (Ozaki et al., 1997), which is a vulnerability gene for CIAS (Douet et al., 2014). In addition, D-serine deregulation is significantly implicated in CIAS (Ma et al., 2019). D-serine, a co-agonist of NMDAR on the glycine binding site (D'Angelo et al., 1990), is oxidized by D-amino acid oxidase (DAO/DAAO), which can regulate the NMDAR function via D-serine breakdown. DAAOs are expressed mainly in cerebellum with little expression in the frontal cortex (Benzel et al., 2008; Jagannath et al., 2017).

In SZ subjects, a reduced expression of the monomeric form of mGluR5 was specifically revealed in the lateral cerebellum and associated with mood disorders in SZ (Fatemi et al., 2013; Matosin et al., 2014; Fatemi and Folsom, 2015).

Moreover, histological alterations were observed in cerebellar slices in a ketamine-induced SZ model in mice; the changes were seen mostly in neurodegenerating cerebellar areas, particularly in PCs showing apoptosis with pyknotic nuclei, irregular dark cytoplasm, and wide interstitial spaces around the cells. This effect was reversed in groups treated with Carpolobia lutea G. Don extract and clozapine (Omeiza et al., 2023). Notably, the recovery of tissue damage was associated with mitigation of positive, negative, and cognitive symptoms.

#### 3.2.5 The cerebellar GABAergic system

GABA is the main inhibitory neurotransmitter in the cerebellum and the entire brain (Section 2.1). In the cerebellum, GABA helps regulate and balance neural activity, contributing to motor control, coordination, and cognitive functions. It was hypothesized that, in the cerebellum, the effectiveness of the GABAergic inhibitory system might be reduced in SZ to counterbalance NMDAR hypofunction (Yeganeh-Doost et al., 2011) contributing to cognitive impairment (Piras et al., 2019). The cellular density of GABAergic Purkinje inhibitory neurons in cerebellum is decreased in psychotic patients (Maloku et al., 2010).

Early findings from clinical and preclinical trials showed alterations in cerebellar GABA signaling in SZ patients. The mRNA and protein levels of GABA synthesizing enzymes GAD67 and reelin, which are expressed in GABAergic interneurons, were downregulated (Guidotti et al., 2000; Fatemi et al., 2005; Bullock et al., 2008). Like other neuromodulators, the alterations were not limited to receptor function and expression but also involved GABA levels. A higher concentration of cerebellar GABA was detected in SZ patients, and this was associated with lower phonemic fluency and a reduced number of switches between subcategories compared to healthy subjects (Piras et al., 2019). GAD56 and the presynaptic GABA transporter GAT-1 were also reduced along with PC density (Bullock et al., 2009; Maloku et al., 2010).

GABA_A_R containing α6 subunits (α6GABAARs) are located at cerebellar GoCs-GrCs synapses and extra-synaptic sites, where they regulate the precision of inputs required for cerebellar timing (Mapelli et al., 2014; Mapelli J. et al., 2022). These receptors have an impact on motor activity and are involved in cognitive processing and adequate responses to external stimuli in the cerebellum, eventually implicated in CIAS (Lee et al., 2022). The α6GABAARs were upregulated in post-mortem cerebellar tissues and in a rat model induced by PCP (Bullock et al., 2009).

Dysfunction in cerebellar GABAergic interneurons leads to reduced synchronization across brain regions, affecting cortical information processing (Yeganeh-Doost et al., 2011). The specific impairment of subsets of cerebellar GABA-expressing interneurons in SZ (Piras et al., 2019; Lee et al., 2022) could disrupt the coordination between cerebellum and cortex contributing to neuropsychological deficits (Piras et al., 2019).

### 3.3 Cerebellar dysconnectivity and CIAS

**Figure 6:**
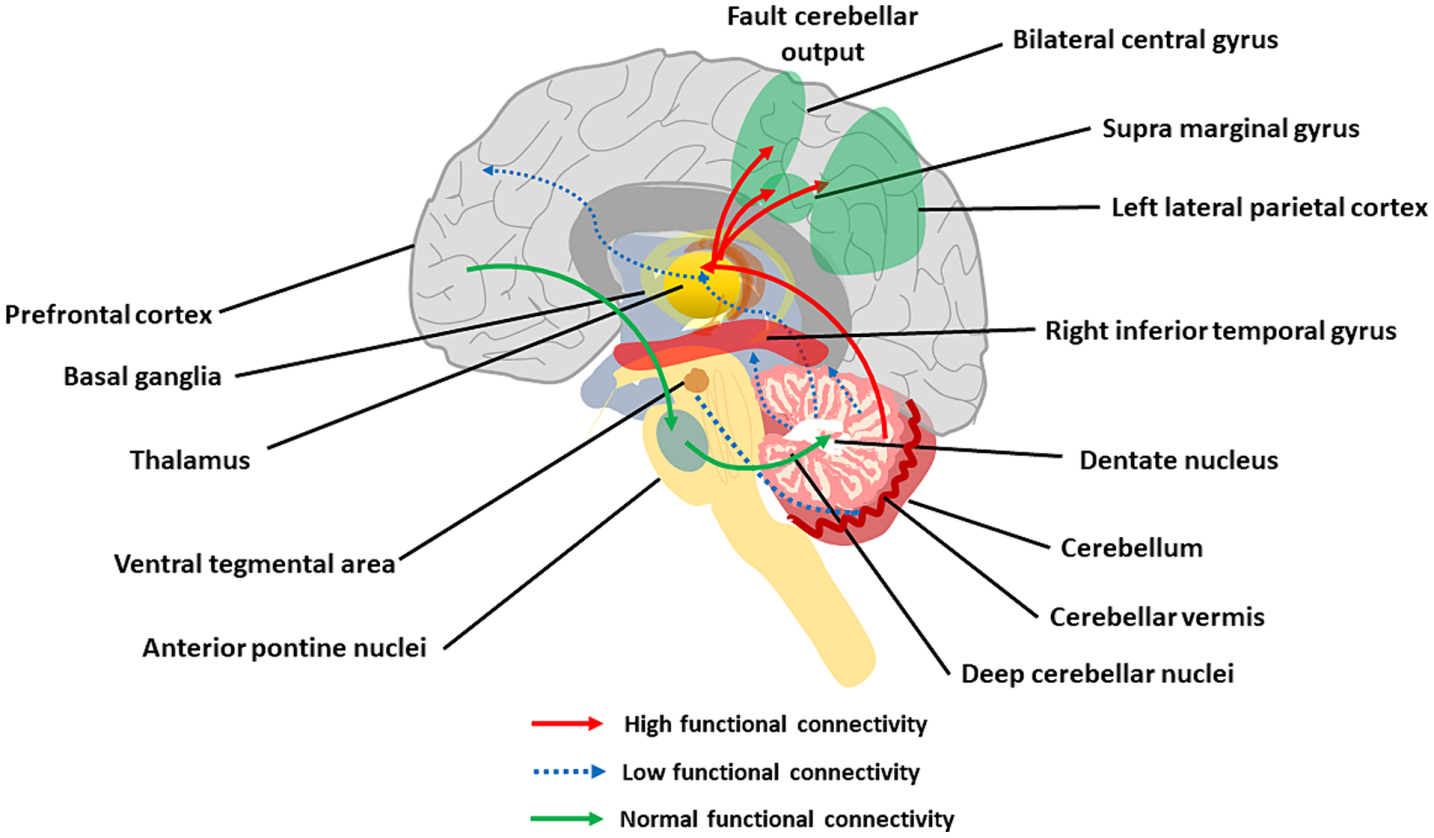
The main aspects of cerebellar dysconnectivity in SZ. In the scheme, different cerebellar projections show either increased (red arrows), decreased (blue arrow), or normal (green arrows) functional connectivity. Note the reduced functional connectivity with the prefrontal cortex and inferior temporal gyrus, the reduced functional connectivity with VTA, and the increased functional connectivity with the parietal cortex (see text for details).

The cerebellar cortical circuit is illustrated in Figure 3A. PCs integrate signals from the IO-*cf* and the mf-GrC-pf pathways, and project to DCN neurons which, in turn, projects back to various brain regions (D'Angelo and Casali, 2012). This way, the cerebellar cortex forms intricate connections with the cerebral cortex, basal ganglia and VTA that are core to CIAS and SZ. The disruption in cerebellar network communication, tied to deficiencies in dopamine, glutamate, and GABA transmission, has been indeed proposed to explain reduced connectivity in SZ patients (Giersch et al., 2016, Katz Shroitman et al., 2023; Figure 6).

#### 3.3.1 CTCC loops and cognitive dysmetria

The cerebellum forms *extensive connections with the forebrain* via *DCN and thalamus* generating cerebello-thalamo-cortical circuits (CTCCs) that are thought to underly cognitive and affective functions (Schmahmann, 2016; Ribeiro and Sherrard, 2023). Functionally, the cerebellar and cerebral systems work in concert to refine the timing of neural operations (Castellazzi et al., 2014, 2018; Palesi et al., 2015, 2017, 2018; Bohne et al., 2019; Fujita et al., 2020; Pisano et al., 2021; Li Y. et al., 2022) (see Supplementary material). Through the thalamus, the cerebellum is implicated in coordinating the coherence of oscillations between cerebral cortical structures (Popa et al., 2014; Gambosi et al., 2023; Heck et al., 2023). The cerebellum, as part of the CTCC, performs an error-detection duty and works as a modulator of cognitive information acquired from the cortex (Bang et al., 2018). SZ was early hypothesized to arise from a disrupted CTCC communication impairing the error detection function of the cerebellum (Andreasen et al., 1998; Andreasen and Pierson, 2008; D'Angelo and Casali, 2012), and a wealth of studies have recently focused on abnormal CTCC connectivity as a core pathology of SZ as well as other psychiatric disorders (Okugawa et al., 2004; Koch et al., 2010; Bang et al., 2018; Hanaie et al., 2018; Wang et al., 2018; Ding et al., 2019; Kim et al., 2021; Park et al., 2021). Lesions of the posterior cerebellum have been related to cognitive dysmetria and CIAS (Yeruva et al., 2021). A seminal work revealed the functional connectivity of DN with whole-brain and its association with cognitive impairments and other psychotic symptoms in patients with drug-naïve and first-episode SZ (Xie et al., 2021). The increased connectivity of DN with the bilateral postcentral gyrus and decreased connectivity of DN with the right inferior temporal gyrus and regional cerebellum (e.g., Vermis IV, V, and Crus I) were correlated with CIAS. The other hub of the CTCC is the thalamus, and altered functional connectivity between cerebellar hemispheres, mediodorsal nucleus, and lateral geniculate nucleus of the thalamus was reported in SZ (Collin et al., 2011; Liu et al., 2011; Chen et al., 2013; Anticevic et al., 2014; Barch, 2014).

Both in first-episode and chronic SZ patients, altered cerebellar functional connectivity in *RS* fMRI was observed with broad cerebral regions, including association networks, the sensorimotor network, the limbic network, basal ganglia network, and the DMN (Liu et al., 2011; Chen et al., 2017; Guo et al., 2018; Zhuo et al., 2018; Xie et al., 2021; Feng et al., 2022). Critical connector hubs were identified using voxel-based analysis in the cerebellum, midbrain, thalamus, insula, and calcarine sulcus, with connectivity to multiple RS networks affected in SZ (Yamamoto et al., 2022). These findings were supported by cognitive *task-dependent fMRI*, in which SZ patients showed significantly increased connectivity between the cerebellum and left lateral parietal cortex compared to healthy participants (King et al., 2023). Reduced blood flow in the CTCC during cognitive tasks in SZ was related to deficits in cerebellar inhibition of the DCN (Daskalakis et al., 2005). The strength of functional connectivity between the cerebellum and lateral parietal regions, such as the postcentral gyrus and supramarginal gyrus, was associated with negative symptoms, including socio-cognitive dysfunctions and cognitive decline in SZ (Guo et al., 2018; Brady et al., 2019; Park et al., 2021; Choi et al., 2023).

#### 3.3.2 Cerebellar connectivity with basal ganglia

The cerebellum sends monosynaptic glutamatergic projections to dopaminergic and non-dopaminergic neurons of substantia nigra pars compacta (Washburn et al., 2024). Moreover, the cerebellum and striatum communicate with the thalamus and cortex via monosynaptic and polysynaptic connections, producing cortico-striatal-thalamic-cerebellar (CSTC) loops. Associative CSTC subdivisions showed consistent brain-wide bi-directional changes in SZ, hyperconnectivity with sensory cortices, and hypoconnectivity with association cortex. Such alterations were strongly related to cognitive impairment (Ji et al., 2019). A study of resting-state networks in SZ patients showed increased functional connectivity in the DMN associated with decreased connectivity in the cerebellar network (Rong et al., 2023). Connectivity of the cerebellum with basal ganglia and regions involved in visual, sensorimotor processing and reward was also altered (Yoshida et al., 2022).

#### 3.3.3 Cerebellar connectivity with VTA

The cerebellum is connected to and transmits direct stimulatory signals to the VTA, a brain region responsible for the elaboration of rewarding experiences. Optogenetic activation of the cerebellum-VTA connections led to a sense of reward. In the three-chambers social task, these connections become more active when the animal engages with the social chamber during exploration. These data define a major, previously unappreciated role of the cerebellum in controlling the reward circuitry and social behavior, indicating that the cerebellum may mediate SZ symptoms through abnormal connections with the midbrain dopamine brain regions, such as VTA (Carta et al., 2019). Interestingly, recent RS fMRI imaging findings from first-episode SZ patients showed decreased static and dynamic functional connectivity of VTA and substantia nigra pars-compacta to cerebellar vermis (lobules VII and IX), thalamus, striatum, prefrontal lobe, and cingulate gyrus (Xue et al., 2023).

#### 3.3.4 Cerebellar neurodevelopment and neuroinflammation

The cerebellum fetal development endures during childhood and influences the postnatal maturation of multiple cortical regions (Wang et al., 2014). Alterations in this process might, in turn, impact on SZ. Indeed, a combined volume reduction in cerebellum (lobules I–V, VIII) and sensorimotor cortex are associated with increased SZ externalizing symptoms (Miquel et al., 2019). Moreover, reduced grey matter volumes in cerebellum and functionally coupled cortical regions are associated with psychiatric symptoms in mid-childhood (Hughes et al., 2023). Interestingly, altered functional connectivity between cerebellum and medial PFC in SZ patients was linked to high childhood trauma scores (Dauvermann et al., 2021). Moreover, increased connectivity between left lateral parietal cortex and cerebellum was correlated with low-grade systemic inflammation and high plasma IL-6 level, higher childhood neglect, and increased DMN connectivity (King et al., 2023). Therefore, the main genetic and epigenetic factors of SZ may also act on the cerebellum driving a cascade of effects impacting the pathogenesis of the disease.

## 4 Summary, conclusions, and perspectives

### 4.1 Summary and key findings

Since the original proposal for the cerebellar involvement in SZ 25 years ago (Andreasen et al., 1998; Andreasen and Pierson, 2008), a large body of evidence has accumulated showing that the schizophrenic brain exhibits various abnormalities in most brain regions controlling cognitive processing (Karlsgodt et al., 2010; Zhao et al., 2018; Sone et al., 2022; Javitt, 2023). On one hand, the cerebral cortex and forebrain regions have revealed alterations in micro- and macro-structure, development, neurotransmission, plasticity, and connectivity. On the other, the hypothesis of the involvement of the cerebellum in SZ is gaining credit. The cerebellum is involved in multiple aspects of cognitive processing (Schmahmann, 2016; D'Angelo, 2018; D'Angelo, 2019; Schmahmann, 2019; Jacobi et al., 2021; Ciapponi et al., 2023; Nguyen et al., 2023) and is connected functionally and anatomically to brain regions that are core domains of CIAS, such as PFC, basal ganglia, and VTA. Cerebellar alterations can either be primary (genetic and epi-genetic) or secondary (compensatory) in origin and emerge on different scales (Figures 3–6):

1. Reduced cerebellar volume, more accentuated in specific areas, reflecting decreased grey matter and white matter thickness.
2. Microcircuit and cellular alterations, including reduced cell density (GrC, PC, and inhibitory interneuron), reduced PC dendritic branching, altered synaptic vesicular transport, and increased connectivity at climbing fiber/PC synapses.
3. Reduced connectivity with other brain structures, including PFC, basal ganglia, and VTA.
4. Reduced functional activation of specific areas during cognitive tasks.
5. Dopaminergic hypofunction, serotonergic unbalance, glutamatergic and GABAergic dysfunction.

A potential explanation of this broad set of alterations is that any changes in brain circuits bring about both direct effects and compensatory responses in various system components, which then reverberate across scales. Altered bidirectional connectivity in psychosis may stem from neurodevelopmental disruptions or compensatory mechanisms, influenced by neurotransmitter systems abnormalities. Dopaminergic dysregulation may disrupt cerebellar E/I balance and dopaminergic projections to cortical regions, while upregulated serotonin receptors promote synaptic pruning and plasticity, possibly leading to hyperconnectivity. Glutamate and GABA elevation in the cerebellum might influence hyperconnectivity by modulating excitatory and inhibitory neurotransmission thus inducing plasticity and connectivity. As a result, these micro-scale modulations of neuronal activity shift network dynamics in response to ongoing demands. These alterations, occurring during neurodevelopment and persisting into adulthood, may disrupt normal connectivity patterns, contributing to psychosis manifestation and progression. This cascade of effects puts cerebellar alterations at the core of the extended brain dysfunction characterizing CIAS.

### 4.2 Cerebellar therapeutic targeting

The cerebellum may provide a promising target for innovative SZ treatments (Parker et al., 2014; Cao and Cannon, 2019; Hua et al., 2022). The specific expression of certain synaptic receptor subtypes (e.g., D1R, and NR2C/DAAO, mGluR5, GABAa6,) in the cerebellar circuit might be exploited.

Cerebellar D1R is a promising therapeutic target for CIAS going beyond the more common D2R antagonists (Goldman-Rakic et al., 2004). D1R agonists were found to enhance blood oxygenation level-dependent (BOLD) signals in the cerebellum in addition to striatum, thalamus, and PFC, while D1R antagonists did the opposite (Kimura et al., 2023). This observation suggests revisiting the Weinberger’s view that D1Rs are principally located in PFC where they are hypo-activated causing negative symptoms (Weinberger, 1987; Slifstein et al., 2015; Rao et al., 2018; McCutcheon et al., 2020), by integrating cerebellar D1R hypofunction as a potential cause of CIAS.

Cerebellar NMDA receptors may be targeted using DAAO antagonists that exploit D-serine sensitivity (Kölker, 2018). Luvadaxistat, a potent DAAO inhibitor, is being developed for the treatment of CIAS and was recently tested with some success in SZ patients (O'Donnell et al., 2023).

Cerebellar α6GABA_A_Rs may be targeted by selective positive allosteric modulators, which proved to alleviate positive, negative, and cognitive impairment in SZ in preclinical studies and rescued PPI by attenuating GrG activity (Chiou et al., 2018; Lee et al., 2022; Sieghart et al., 2022).

Moreover, invasive and non-invasive neuromodulation methods have been proposed to specifically target the cerebellum (Heath et al., 1980, 1981; Aparício et al., 2016; Laidi et al., 2020; Hua et al., 2022; Pilloni et al., 2022). TMS and tDCS can modulate PCs and then regulate DCN activity (Koch, 2010) and neural plasticity (D'Angelo et al., 2016). In preclinical studies, low-intensity rTMS caused PC dendrite and spine changes (Morellini et al., 2015) and tDCS regulated the PC output (Grimaldi et al., 2014; Pope and Miall, 2014; van Dun et al., 2016). Interestingly, the effectiveness of these stimulations extended beyond the local circuit to extracerebellar networks causing, for example, changes in dopamine release (Fonteneau et al., 2018). Improvements in SZ cognitive symptoms were detected following cerebellar stimulation in different clinical trials (Escelsior and Belvederi Murri, 2019). For example, rTMS on posterior cerebellum could boost functional connectivity of the cerebellar-prefrontal circuitry ameliorating clinical symptoms (Brady et al., 2019; Singh et al., 2019; Basavaraju et al., 2021; Chauhan et al., 2021). It has been proposed that rTMS corrects alterations in error processing, which depend on information transfer and integration in the cerebellar-cortical circuitry (Hengyi Cao and Cannon, 2019). Interestingly, optogenetic stimulation of thalamic synaptic terminals of lateral cerebellar projection neurons in a rodent model of SZ-related frontal dysfunction rescued timing performance as well as medial frontal activity (Parker et al., 2017), suggesting that pathway-specific targeting is needed to improve the specificity of physical interventions on the cerebellum.

### 4.3 Open issues

There is still a large gap in our understanding of cerebellar involvement in SZ, especially concerning the initial alterations and their subsequent development, propagation, and compensation.

First, although abnormalities in the cerebellar neurotransmitter system have been documented, the intricate interconnections among these systems await elucidation. Open issues concern potential alterations in E/I balance and synaptic plasticity (Mapelli L. et al., 2022), whose exploration would require physiological investigations in animal models of SZ (Section 2 in Supplementary material). Of special interest is understanding how cerebellar hypo-dopaminergic function might influence the glutamatergic and GABAergic systems and how this, in turn, modulates cerebellar E/I balance and determines the PC output. This is also true for cerebellar serotonin shortages observed in CIAS, and a full revisitation is needed for the cholinergic system (Zhang et al., 2016).

Secondly, several questions regarding how cerebellar circuits operate in the context of CIAS-related circuits remain open. The main one is whether the universal cerebellar transform (Ito, 2008; D'Angelo and Casali, 2012) is altered and how, in turn, this impacts cognitive performance in SZ. Related to this is the differentiation of activity and neuromodulation among specific cerebellar regions (Ciapponi et al., 2023). This is particularly pertinent to the posterior lobules, which hold a pivotal role in cognitive processing.

Thirdly, it is not clear how shortages in cerebellar connectivity with other brain regions, including cerebral cortex, basal ganglia, and VTA, impact SZ. In turn, cerebellar dysconnectivity is related to neurodevelopment changes. Dysconnectivity may be, again, either a primary or a secondary event in SZ pathogenesis and bring about plastic changes that modify brain functions at system level.

### 4.4 Perspectives

Cognitive and negative symptoms are principal contributors to disability in SZ, but they are yet poorly treated by current therapies. The cerebellar involvement in CIAS (Section 4.1) is disclosing a promising target for therapeutic interventions (see Section 4.2). Thus, addressing gaps in knowledge is necessary to achieve a more comprehensive understanding of CIAS and its underlying mechanisms (see Section 4.3). In addition to MRI and electrophysiological recordings in humans, the precise analysis of neuronal activity and synaptic transmission and plasticity in animal models is needed to explain SZ-related alterations in connectivity, E/I balance, and synaptic plasticity, as well as in the GABAergic, glutamatergic, dopaminergic, serotonergic, and cholinergic systems of the cerebellum. Computational models can then be used to further understand the complex and heterogeneous nature of this disorder (Amunts et al., 2022; D'Angelo and Jirsa, 2022; Monteverdi et al., 2022, 2023) paving the way for precise and personalized therapeutic approaches, especially in treating cognitive shortages.

## Author contributions

PF: Conceptualization, Supervision, Visualization, Writing – original draft, Writing – review & editing. DP: Writing – original draft, Writing – review & editing. FP: Writing – original draft, Writing – review & editing. ED’A: Conceptualization, Funding acquisition, Supervision, Visualization, Writing – original draft, Writing – review & editing.
