# Supplementary material for: New clues for the role of cerebellum in schizophrenia and the associated cognitive impairment
Source: Front Cell Neurosci. 2024 May 10;18:1386583. doi: 10.3389/fncel.2024.1386583 (PMC11116653; doi:10.3389/fncel.2024.1386583)
Supplement: Supplementary file 1 [file Data_Sheet_1.PDF]

Supplementary Material

1 Supplementary Information

1.1 Cerebellum: from Motor to Cognitive Function

The cerebellum has long been known as a major structure for motor function: in the presence of cerebellar lesion, or under cerebellar malfunction, alterations in movement and coordination in humans and animals are observed (Glickstein, Strata et al. 2009). The cerebellum intervenes in high-level motor processes, such as sensorimotor synchronization, oculomotor control, and motor learning, by improving performance by trial-and-error learning (Manto 2003). It regulates the timing of movement making use of implicit memory performing the operation called 'sensory prediction'(Ivry and Spencer 2004). The cerebellum receives motor commands from the primary motor cortex and predicts the sensory feedback. The prediction is then compared with the actual feedback from sensory inputs. In general, the cerebellar network expresses three main functional properties: (I) An adaptive filter, that is, the ability to modify its internal parameters through learning. This operation is carried out in both the granular and molecular layers. The master actors are mossy fiber (mf), parallel fiber (pf), and Purkinje cells (PCs) (Dean and Porrill 2008, Dean, Porrill et al. 2010, Dean and Porrill 2011, Wilson, Anderson et al. 2019). (II) Input pre-processing, which is mainly performed by the granular cell (GrC) layer. This allows for pattern separation by exploiting synaptic connectivity, spike thresholding, multimodal integration, and broad feedback inhibition (D'Angelo and Casali 2012, D'Angelo and Casali 2012, Cayco-Gajic and Silver 2019, Casali, Tognolina et al. 2020). (III) An instructive signal that leads to appropriate adjustments in the adaptive filter in the presence of an error (i.e., the difference between the predicted and the actual movement) during supervised learning. Such signals are conveyed by PCs complex spikes that are driven by climbing fibers (cfs). The PC can also instruct plasticity and influence network response by enhanced firing at rest (Raymond and Medina 2018, Silva, Ramírez-Buriticá et al. 2022). Acquiring new motor skills typically entails transitioning from a controlled to an automatic processing mode, where the initial movement requires problem-solving and attention and becomes progressively more efficient, stereotyped, and significantly less dependent on attention (Koziol and Lutz 2013).

Although the cerebellar deficit impacts motor activity, robust evidence now indicates that the cerebellum is also heavily involved in cognitive processing, including working memory, language skills, motion perception, and emotional processing (D'Angelo 2018, D'Angelo 2019, Schmahmann 2019, Jacobi, Faber et al. 2021, Ciapponi, Li et al. 2023, Nguyen, Thomas et al. 2023). The cerebellar system is entrained in large-scale brain networks during cognitive processes (Castellazzi, Bruno et al. 2018). Multiple theories emerged to explain the role of the cerebellum in cognitive processes, some of them as an extrapolation of motor functions. The first common theory is "Dysmetria of thought", which extends the involvement of cerebellum from sensorimotor function to cognition and emotion under a single overarching process, the so called "universal cerebellar transform", that can be applied to all mental functions (Schmahmann 2019). The basis of the theory considers the stereotypical architecture

of the cerebellum, which gains specific function once coupled through topographical connections with other cerebral regions. This theory also implies segregation of functions in specific region of the cerebellum. This regionalization corresponds to patient impairments: anterior lesions cause motor deficits, while posterior lesions lead to cognitive decline (Schmahmann 2019). Human fMRI analysis allows visualization of cerebellar activation during various motor and cognitive tasks generating a functional topography map of the cerebellum (see supplementary Figure 1) (Stoodley and Schmahmann 2009, Stoodley, Valera et al. 2012). Multiple forms of synaptic and non-synaptic plasticity have been observed in cerebellar networks, substantiating and extending the "motor learning theory" of Marr (Marr 1969). Supervised learning plays a crucial role in PCs by exploiting feedback on system performance to adjust internal configurations and enhance future performance. This implies that the cerebellum establishes an internal prediction mechanism, which is then compared with sensory feedback to guide proactive behaviours (Hull 2020). Unsupervised learning occurs at other synapses, e.g., in the granular layer. In aggregate, nowadays there is robust evidence that the cerebellum, apart from its role in motor control, is crucially involved in a wide spectrum of cognitive and affective functions. Clinical and preclinical studies, together with evidence from anatomical studies and advanced neuroimaging, have provided a deeper understanding and insights into the particular features and clinical relevance of cerebellar involvement in cognitive functions (Jacobi, Faber et al. 2021, Bègue, Elandaloussi et al. 2023). Interestingly, the meta-level hypothesis that was developed to connect the UCT to the multiple functions the cerebellum subserves in the motor and emotional/cognitive domains, identifies learning, timing, and prediction as the kernels of neural network computation that give rise to working-memory and attention capabilities (D'Angelo and Casali, 2013). These are indeed the same cognitive domains affected in SZ (see section 2), generating a solid basis for the implication of the cerebellum in SZ.

1.2 An Overview of the Cognitive Cerebellum

Anatomical observations from the monkey and human brains have revealed functional and structural connectivity between cerebellum and other brain regions organized in close loop (Schmahmann and Pandya 1997, Strick, Dum et al. 2009, D'Angelo and Casali 2012, Palesi, Tournier et al. 2015, Palesi, De Rinaldis et al. 2017). D'Angelo and Casali hypothesised that the cerebellum operates as a general co-processor, relying on hubs to which various modules are interconnected, affecting sensorimotor systems and high cognitive functions (D'Angelo and Casali 2012). The cerebellum has a remarkable diversity of molecular, cellular, and circuit mechanisms, embedded in a dynamic, recurrent circuit architecture on multiple scales (De Zeeuw, Lisberger et al. 2021). On the microscale (neuronal circuits), the cerebellum consists of a well-organized loop of neurons, interneurons, and fibres connected anatomically and functionally (see Figure 3) (D'Angelo and Casali 2012). On the mesoscale, the cortex and grey matter are tightly folded into layers with white matter underneath and are tightly interconnected with the deep cerebellar nuclei (DCN) and inferior olive (Apps and Garwicz 2005). On the macroscale, the afferent and efferent cerebellar pathways take part in cerebello-thalamo-cerebro-cortical circuits (CTCCs). Generally, there is a bidirectional connection between cerebellum and cerebral cortex, which involves cerebral cortical regions that are either motor or associative. Resting-state networks and functional connectivity of the cerebellum for motor, sensory, and cognitive tasks

help to shed light on the networks underlying affective-cognitive function (D'Angelo and Casali 2012, Castellazzi, Palesi et al. 2014, Uyy, Suica et al. , Habas 2021). Different cerebellar regions take part to several networks, including the default mode network, salience network, and attention network, enabling the cerebellum to supervise and coordinate brain activities subtending thinking and emotion, possibly by calculating internal models. In summary, understanding the involvement of the cerebellum in cognitive impairment can provide valuable insights into the neurobiology of CIAS in SZ and, potentially, suggest effective diagnostic and treatment approaches. Several experimental and neuroimaging investigations have indicated that the posterior lobe and the vermis of the cerebellum exhibit extensive connectivity with the association regions of the cerebral cortex and limbic system (Bostan and Strick 2018, Carta, Chen et al. 2019). Utilizing its wide array of capabilities, the cerebellum can accurately predict and control events by acting as a predictive time controller (D'Angelo and Casali 2012). The cerebellum can predict events at a low-level complexity, such as timing, sensory prediction, and sequence learning (Moberget, Karns et al. 2008, D'Angelo and Casali 2012). These processing primitives can be applied to complex behavioural operations including high-level cognitive functions (D'Angelo and Casali 2012). Recent research suggests that alterations in the structure of the cerebellum may contribute to cognitive decline. Indeed, multiple investigations have found comparable modifications in several psychiatric diseases, including SZ (Hanaie, Mohri et al. 2018, Wang, Zhong et al. 2018, Kim, Jung et al. 2021, Feng, Zheng et al. 2022) (see below).

1.3 Topography of the Cognitive Cerebellum

The cerebellum, which accounts for 10% of total brain volume, 80% of total brain surface area, and contains more than half of the brain neurons altogether, is a core component of the Central Nervous System and contains the second main cortex of the brain. Evolutionary, the cerebellum is the brain's oldest cortical structure, first forming in eel-like organisms 300 million years ago. Anatomically, it is divided into three layers: granular, Purkinje, and molecular. This division is determined by three factors: midline positioning, fissures, and evolutionary development. Within these layers, there exist subzones or micro complexes that together form a fractured somatotopy or mosaic arrangement (Apps and Garwicz 2005, Lara-Aparicio, Laureani-Fierro et al. 2022, Ciapponi, Li et al. 2023). Since the “cognitive dysmetria” model of SZ was introduced, the cerebellum has become widely implicated in CIAS (Andreasen, O'Leary et al. 1996). A new evolutionary discovery linked the higher presence of early fetal Purkinje cells in humans to potentially expanded neuronal progenitor pools of the cerebellum, likely influencing granule cell generation, and aligning with neocortex expansion. This process, during evolution, could contribute to the emergence of complex cognitive functions in humans (Sepp, Leiss et al. 2023). CIAS refers to the failure of the cerebellum to effectively regulate and synchronize diverse information transmitted from the cerebral cortex, resulting in inaccurate cognition and behaviours (Andreasen and Pierson 2008). Generally, the majority of the cerebellar cortex is not primarily involved in motor functioning. Indeed, the motor-related parts are relatively minor and restricted in their extent and conserved across subjects (Diedrichsen and Zotow 2015, Stoodley and Schmahmann 2018).

The cerebellum consists of 10 lobules: the anterior lobe includes lobules I-V, while the posterior one includes VI-IX, and lobule X is the folecculonodular lobe. Moreover, lobule VII can be further

subdivided into Crus I, Crus II, and VIIb (Figure S1). The motor-related lobules are those of the anterior part, with some extension to the posterior VI and VII lobules. Under resting condition, imaging studies showed a triple representation of cerebellar cortex related to cognitive functions (Buckner, Krienen et al. 2011). There are two inverted representations in the posterolateral cerebellar hemispheres (attentional/executive network-related areas-default-mode network-default-mode network-attentional/executive network), and a third representation in lobules IX and X that overlaps with the cerebellar vestibular system. Interestingly, a recent study found a third somatomotor representation in the posterior vermis, supporting the concept that the cerebellum has three sets of roughly homotopic representations of the entire cerebrum (Saadon-Grosman, Angeli et al. 2022). For more details on anatomical and functional human cerebellum topography, see the review (van Es, van der Zwaag et al. 2019, Guell and Schmahmann 2020) and for rodents, see the review (Ciapponi, Li et al. 2023). Structural neuroimaging data from the Healthy Brain Network reveals a strong association between the structure of the cerebellar Crus II and Lobule X and cognitive flexibility (the ability to switch rapidly between mental states and tasks) (Bègue, Elandaloussi et al. 2023). Notably, the cerebellar grey matter volume is linked to cognitive flexibility abilities in a dimensional across-diagnostic categories manner. Conversely, cognitive stability, the ability to maintain stable cognitive representations, is also important for consistent and reliable behaviour, and requires less task switching and more working memory capacity (Bègue, Elandaloussi et al. 2023).

2 Animal Models for SZ and CIAS: Preclinical Studies

Rodent models that recapitulate the aetiologies, brain diseases, and behavioural abnormalities linked with SZ in humans have made tremendous progress. These models include pharmacological (based on dopamine or glutamate system hypofunction), genetic (based on disorders in genes related to SZ), and neurodevelopmental models (involving damage to relevant brain regions during the fetal or neonatal period of rodents), see review (Białoń and Wąsik 2022). A growing number of preclinical trials revealed the potential implication of cerebellum in cognitive processes, including spatiotemporal navigation through a functional link of cerebellar PCs with hippocampal place cells (Rochefort, Arabo et al. 2011), reward expectation processing encoded by cerebellar GrCs (Wagner, Kim et al. 2017), reward-related reinforcement learning (Sendhilnathan, Semework et al. 2020) through PCs, and modulating reward circuity through a direct monosynaptic connection between DCNs and VTA and social behaviour (Carta, Chen et al. 2019, D'Angelo 2019). Similarly, several animal models have been developed to better understand the cerebellar implication in SZ, particularly, impairments associated with cognitive defects, although there has been relatively little preclinical research focused on cerebellar abnormalities in SZ. However, this hypothesis is gaining attraction and becoming a major focus of investigation. Here, we show relevant evidence that emphasizes the implication of cerebellar dysfunction in CIAS, shedding light on various animal models that were developed for this purpose.

A study using a double-hit stress murine model, a model of SZ induced by exposing the offspring to maternal deprivation and social isolation, pointed out some molecular network alterations observed in SZ that were a consequence of different combinations of stress exposure during the late postnatal developmental phase of the cerebellum (Vera-Montecinos, Rodríguez-Mias et al. 2021). As a result,

this study indicated that the cerebellum is an area vulnerable to accumulating molecular errors induced by stress during early postnatal life in SZ (Vera-Montecinos, Rodríguez-Mias et al. 2021). In a subsequent study, the same model elucidated a reduction in methyl transferase-like protein 7A (METTL7A), a member of the METTL family of methyltransferases, which was found to be altered in different brain regions of SZ patients, particularly in the cerebellar cortex (Vera-Montecinos, Rodríguez-Mias et al. 2021). Specifically, high expression of METTL7A protein level at the end-feet of Bergmann glia and in the contacts of these cells with PCs was observed in SZ models (Vera-Montecinos, Galiano-Landeira et al. 2023). Moreover, it has been evidenced that the alteration in the METTL7A level is associated with cognitive performance (Gong, Yu et al. 2021).

Maternal immune activation (MIA), one of the common strategies to induce SZ animal models, acting during specific gestational timings, has been implicated in increasing schizophrenia risk in offspring (Meehan, Harms et al. 2017). This stimulation causes neuronal connectivity and synaptic pruning dysfunction, which may contribute to the pathological characteristics seen in post-mortem brain of SZ individuals (e.g., decreased cortical grey matter thickness), leading to social deficits (Comer, Jinadasa et al. 2020, Magdalon, Mansur et al. 2020). Neuropathological signs were detected in the offspring of MIA models including deficits in corticogenesis, reduced hippocampal volume, reduced GABA markers and behavioural abnormalities linked to SZ (Kneeland and Fatemi 2013, Choudhury and Lennox 2021). In a MIA female model, cerebellar white matter volume was reduced (Casquero-Veiga, Lamanna-Rama et al. 2023). Moreover, earlier studies observed a localized deficit in PCs in lobule VII of the cerebellum, in adult and P11 offspring, along with delayed migration of GrCs in lobules VI and VII(Shi, Smith et al. 2009). Cerebellar hyperplasia and alterations in motor and social behaviours were observed in MIA mice and that was linked with altered neuronal survival or impaired normal programmed cell death of cerebellar PCs (Aavani, Rana et al. 2015).

Pharmacological animal models, such as PCP, a widely used strategy to induce SZ–like symptoms, are based on the NMDA hypothesis and lead to the development of SZ models using both acute and chronic administration of PCP to study the pathophysiology of this illness (Jones, Watson et al. 2011). Postnatal administration of PCP in rodents has been tested and shown to produce a wide range of behavioural alterations in the adult, including spatial memory deficits and a deficit in social novelty discrimination (Wang, McInnis et al. 2001, Nakatani-Pawlak, Yamaguchi et al. 2009, Clifton, Morisot et al. 2013). A recent study elucidated that neonatal application of PCP induces afferent-specific synaptic deficits in cerebellar PCs, and that leads to long-lasting alteration in cf/PC connectivity (Veleanu, Urrieta-Chávez et al. 2022). Methamphetamine and PCP mouse models, two models based on hyperdopaminergic and hypo glutamatergic hypotheses of SZ, respectively, cause disruptions of prepulse inhibition (PPI) of the startle response (Bullock, Bolognani et al. 2009), in which chronic exposure to low levels of PCP in rats mimics the GABAergic alterations reported in the cerebellum of patients with SZ. Instead, sub-chronic neonatal administration of PCP leads to specific long-term synaptic changes in PCs and transient gene expression changes in the cerebellum. Additionally, a pcp2-cre genetic mouse model, a model to study PCs activities, showed that PCs in the posterior vermis drive rapid, bidirectional changes in aggressive and social behaviour (Jackman, Chen et al. 2020). Besides, the concept of cerebellar GABAergic hypothesis in CIAS has been evidenced in seminal work by Lee and colleagues,

in which the administration of positive allosteric modulators of α6GABAARs, a subtype of $GABAA_A$R known to be almost exclusively expressed in GrCs (Gutiérrez, Khan et al. 1996), rescued chronic PCP-induced social withdrawal, cognitive impairment and disrupted PPI. However, the motor impairment in both PCP and methamphetamine-induced SZ mouse models was not affected(Lee, Mouri et al. 2022). Furthermore, in the sub-chronic exposed PCP female rat model, global brain volume reduction was observed, including the cerebellar upper layer, and that drop was associated with novel object exploration (Doostdar, Kim et al. 2019). Additionally, ketamine, at subanaesthetic doses, produces hallucinations, obsession, as well as CIAS (Białoń and Wąsik 2022). In SZ mouse models exposed to ketamine (a non-selective antagonist of NMDAR), increased cerebellar oxidative stress, IL-10, and anti-inflammatory cytokines were observed, and resulted in social behaviour dysfunction. Such insults were reversed by celastrol (an extract from medicinal plants) administration(Schiavone, Tucci et al. 2019).

Regarding genetic rodent models, from which the cerebellar involvement in CIAS were investigated, a cerebellar volume reduction was observed in the Disc1 mouse model (Clapcote, Lipina et al. 2007). Overexpression of Disc1 gene, was found to be strongly linked to cognitive flexibility and social behaviour impairments in SZ- Disc1 rat model (Wang, Chao et al. 2022). However, in water maze tasks, this model showed intact spatial learning and memory, but was deficient in flexible adaptation to spatial reversal learning compared to littermate controls. Consequently, these results indicate that the cerebellum might potentially be implicated in this complication, as the reversal learning task has been found to rely on a network of diencephalic and neocortical cerebellar structures (e.g., lobule VI vermis or hemispheric crus I PCs), and in flexibility of free behaviour (Verpeut, Bergeler et al. 2023). In addition to Disc1, 22q11.2 deletion was also found to be implicated in SZ (Schmitt, Falkai et al. 2023). Indeed, cerebellar volume reduction was revealed in patients with such a genetic alteration, particularly reduction in lobule VII, VIII (Schmitt, Falkai et al. 2023) and in the right cerebellum and inferior temporal gyrus (Frascarelli, Accinni et al. 2023). This evidence was replicated in a 22q11.2 mouse model, in which the whole cerebellar volume was 5% smaller in mutant mice compared to littermate wild type. The affected cerebellar sub-regions include bilateral hemispheric areas (the flocculous and para-flocculus, Crus I dorsal surface, and medial aspects of the anterior lobule), while, in vermis, lobules IV/V, IX and X showed robust volume decreases, along with superior vestibular and vestibulocerebellar nuclei. These cerebellar alterations were associated with CIAS (Ellegood, Markx et al. 2014). Along with volume reduction, the 22q11.2 mouse model showed widespread cortical hypoconnectivity, accompanied by opposing hyperconnectivity in dopaminergic pathways, which was confirmed by graph analysis (Reinwald, Sartorius et al. 2020). Consequently, the combination of cortical hypoconnectivity and dopaminergic hyperconnectivity and reduced cerebellum volume in 22q11.2 deletion models mirrors key neurodevelopmental landscapes of SZ. Besides the cerebellar abnormalities, reduced LTP was observed in hippocampus-medial prefrontal cortex of this model and was associated with defects in several cognitive domains, such as poor cognitive flexibility in the attentional set-shifting task, disparate decision-making choices in the gambling task, impaired short-term spatial memory in the Y-maze task, impaired associative recognition memory, poor prepulse inhibition, and impaired social contact (Tripathi, Spedding et al. 2020). Moreover, Tabata and

colleagues found reduction in the dendritic branches and/or dendritic spine densities of neurons in the medial prefrontal cortex, nucleus accumbens, and primary somatosensory cortex (Tabata, Mori et al. 2023). Along with this, reduced axon innervation of dopaminergic neurons into the prefrontal cortex was observed in the 22q11.2 mice (Tabata, Mori et al. 2023). Moreover, the 16p11.2 microduplication is associated with different neuropsychiatric disorders and exhibits cerebellar abnormalities, reflected in transcriptional dysregulation and microcephaly in affected individuals. Investigation into 16p11.2$^{dp/+}$ mouse models reveals altered cerebellar structure, including mis-localized PCs and reduced ML interneurons in lobule VI, impacting cerebellar learning and conditioned responses, mirroring behaviors observed in humans with 16p11.2 microduplication-associated neuropsychiatric conditions such as SZ and ADHD (Hayes, Halverson et al. 2023). The cerebellar impairments have been detected in other genetic animal models, such as Nrg1 mouse SZ model. Various preclinical studies addressed the neuropathological role of NRG1 protein and related signalling in SZ, focusing on different brain regions, reviewed elsewhere (Białoń and Wąsik 2022). However, the cerebellar Nrg1, a gene that encodes a tropic factor, was not studied, and its association with several neuropsychiatric disorders including SZ (Yang, Si et al. 2003) was not clear till a recent study showed the cerebellar distribution of Nrg1(Ding, Ding et al. 2023). In a Nrg1 knock-in mouse model, the gene was found to be extensively expressed in cerebellar PCs and GABAergic interneurons in the granular layer, while its related receptor ErbB4 was mostly expressed in Bergmann glia cells in the Purkinje layer (Ding, Ding et al. 2023). Human genetic studies have implicated the primate-specific gene locus G72 in SZ (Shevelkin, Ihenatu et al. 2014). In addition, high serum G72 levels were detected in SZ (Akyol, Albayrak et al. 2017). Compared to WT mice, G72Tg mice showed altered expression of proteins involved in myelin-related processes, oxidative stress, and mitochondrial function in the cerebellum, indicating the potential molecular correlates of SZ-like behaviour. Treating defective mice with antioxidant N-acetyl cysteine, a precursor of glutathione, rescued the cognitive deficit, particularly the spatial learning (Otte, Sommersberg et al. 2011).

Overall, while the extent of the cerebellar involvement in the CIAS remains limited, its significance has been demonstrated in several animal models of SZ. Findings from this evidence strongly indicate that the cerebellum plays a crucial role in CIAS. However, further investigation is necessary to address the missing information in the current models and to explore other SZ models that have not been studied. Replicating the cerebellar hypotheses in CIAS, for example, could provide valuable insights. Earlier preclinical models were reviewed thoroughly elsewhere (Shevelkin, Ihenatu et al. 2014).

3 References

Aavani, T., S. A. Rana, R. Hawkes and Q. J. Pittman (2015). "Maternal immune activation produces cerebellar hyperplasia and alterations in motor and social behaviors in male and female mice." Cerebellum **14**(5): 491-505.

Akyol, E. S., Y. Albayrak, N. Aksoy, B. Şahin, M. Beyazyüz, M. Kuloğlu and K. Hashimoto (2017). "Increased serum G72 protein levels in patients with schizophrenia: a potential candidate biomarker." Acta Neuropsychiatr **29**(2): 80-86.

Andreasen, N. C., D. S. O'Leary, T. Cizadlo, S. Arndt, K. Rezai, L. L. Ponto, G. L. Watkins and R. D. Hichwa (1996). "Schizophrenia and cognitive dysmetria: a positron-emission tomography study of dysfunctional prefrontal-thalamic-cerebellar circuitry." Proc Natl Acad Sci U S A **93**(18): 9985-9990.

Andreasen, N. C. and R. Pierson (2008). "The role of the cerebellum in schizophrenia." Biol Psychiatry **64**(2): 81-88.
Apps, R. and M. Garwicz (2005). "Anatomical and physiological foundations of cerebellar information processing." Nat Rev Neurosci **6**(4): 297-311.
Białoń, M. and A. Wąsik (2022). "Advantages and Limitations of Animal Schizophrenia Models." International Journal of Molecular Sciences **23**(11): 5968.
Bostan, A. C. and P. L. Strick (2018). "The basal ganglia and the cerebellum: nodes in an integrated network." Nature Reviews Neuroscience **19**(6): 338-350.
Buckner, R. L., F. M. Krienen, A. Castellanos, J. C. Diaz and B. T. Yeo (2011). "The organization of the human cerebellum estimated by intrinsic functional connectivity." J Neurophysiol **106**(5): 2322-2345.
Bullock, W. M., F. Bolognani, P. Botta, C. F. Valenzuela and N. I. Perrone-Bizzozero (2009). "Schizophrenia-like GABAergic gene expression deficits in cerebellar Golgi cells from rats chronically exposed to low-dose phencyclidine." Neurochem Int **55**(8): 775-782.
Bègue, I., Y. Elandaloussi, F. Delavari, H. Cao, A. Moussa-Tooks, M. Roser, P. Coupé, M. Leboyer, S. Kaiser, J. Houenou, R. Brady and C. Laidi (2023). "The cerebellum and cognitive function: anatomical evidence from a transdiagnostic sample." medRxiv: 2023.2002.2022.23286149.
Carta, I., C. H. Chen, A. L. Schott, S. Dorizan and K. Khodakhah (2019). "Cerebellar modulation of the reward circuitry and social behavior." Science **363**(6424): eaav0581.
Casali, S., M. Tognolina, D. Gandolfi, J. Mapelli and E. D'Angelo (2020). "Cellular-resolution mapping uncovers spatial adaptive filtering at the rat cerebellum input stage." Commun Biol **3**(1): 635.
Casquero-Veiga, M., N. Lamanna-Rama, D. Romero-Miguel, H. Rojas-Marquez, J. Alcaide, M. Beltran, J. Nacher, M. Desco and M. L. Soto-Montenegro (2023). "The Poly I:C maternal immune stimulation model shows unique patterns of brain metabolism, morphometry, and plasticity in female rats." Frontiers in Behavioral Neuroscience **16**.
Castellazzi, G., S. D. Bruno, A. T. Toosy, L. Casiraghi, F. Palesi, G. Savini, E. D'Angelo and C. Wheeler-Kingshott (2018). "Prominent Changes in Cerebro-Cerebellar Functional Connectivity During Continuous Cognitive Processing." Front Cell Neurosci **12**: 331.
Castellazzi, G., F. Palesi, S. Casali, P. Vitali, E. Sinforiani, C. A. Wheeler-Kingshott and E. D'Angelo (2014). "A comprehensive assessment of resting state networks: bidirectional modification of functional integrity in cerebro-cerebellar networks in dementia." Front Neurosci **8**: 223.
Cayco-Gajic, N. A. and R. A. Silver (2019). "Re-evaluating Circuit Mechanisms Underlying Pattern Separation." Neuron **101**(4): 584-602.
Choudhury, Z. and B. Lennox (2021). "Maternal Immune Activation and Schizophrenia–Evidence for an Immune Priming Disorder." Frontiers in Psychiatry **12**.
Ciapponi, C., Y. Li, D. A. Osorio Becerra, D. Rodarie, C. Casellato, L. Mapelli and E. D’Angelo (2023). "Variations on the theme: focus on cerebellum and emotional processing." Frontiers in Systems Neuroscience **17**.
Clapcote, S. J., T. V. Lipina, J. K. Millar, S. Mackie, S. Christie, F. Ogawa, J. P. Lerch, K. Trimble, M. Uchiyama, Y. Sakuraba, H. Kaneda, T. Shiroishi, M. D. Houslay, R. M. Henkelman, J. G. Sled, Y. Gondo, D. J. Porteous and J. C. Roder (2007). "Behavioral phenotypes of Disc1 missense mutations in mice." Neuron **54**(3): 387-402.
Clifton, N. E., N. Morisot, S. Girardon, M. J. Millan and F. Loiseau (2013). "Enhancement of social novelty discrimination by positive allosteric modulators at metabotropic glutamate 5 receptors: adolescent administration prevents adult-onset deficits induced by neonatal treatment with phencyclidine." Psychopharmacology (Berl) **225**(3): 579-594.

Comer, A. L., T. Jinadasa, B. Sriram, R. A. Phadke, L. N. Kretsge, T. P. H. Nguyen, G. Antognetti, J. P. Gilbert, J. Lee, E. R. Newmark, F. S. Hausmann, S. Rosenthal, K. Liu Kot, Y. Liu, W. W. Yen, B. Dejanovic and A. Cruz-Martín (2020). "Increased expression of schizophrenia-associated gene C4 leads to hypoconnectivity of prefrontal cortex and reduced social interaction." PLoS Biol **18**(1): e3000604.

D'Angelo, E. (2018). Chapter 6 - Physiology of the cerebellum. Handbook of Clinical Neurology. M. Manto and T. A. G. M. Huisman, Elsevier. **154:** 85-108.

D'Angelo, E. (2019). "The cerebellum gets social." Science **363**(6424): 229-229.

D'Angelo, E. and S. Casali (2012). "Seeking a unified framework for cerebellar function and dysfunction: from circuit operations to cognition

" Front Neural Circuits **6**: 116.

D'Angelo, E. and S. Casali (2012). "Seeking a unified framework for cerebellar function and dysfunction: from circuit operations to cognition." Front Neural Circuits **6**: 116.

De Zeeuw, C. I., S. G. Lisberger and J. L. Raymond (2021). "Diversity and dynamism in the cerebellum." Nat Neurosci **24**(2): 160-167.

Dean, P. and J. Porrill (2008). "Adaptive-filter models of the cerebellum: computational analysis." Cerebellum **7**(4): 567-571.

Dean, P. and J. Porrill (2011). "Evaluating the adaptive-filter model of the cerebellum." J Physiol **589**(Pt 14): 3459-3470.

Dean, P., J. Porrill, C. F. Ekerot and H. Jörntell (2010). "The cerebellar microcircuit as an adaptive filter: experimental and computational evidence." Nat Rev Neurosci **11**(1): 30-43.

Diedrichsen, J. and E. Zotow (2015). "Surface-Based Display of Volume-Averaged Cerebellar Imaging Data." PLoS One **10**(7): e0133402.

Ding, C.-Y., Y.-T. Ding, H. Ji, Y.-Y. Wang, X. Zhang and D.-M. Yin (2023). "Genetic labeling reveals spatial and cellular expression pattern of neuregulin 1 in mouse brain." Cell & Bioscience **13**(1): 79.

Doostdar, N., E. Kim, B. Grayson, M. K. Harte, J. C. Neill and A. C. Vernon (2019). "Global brain volume reductions in a sub-chronic phencyclidine animal model for schizophrenia and their relationship to recognition memory." J Psychopharmacol **33**(10): 1274-1287.

Ellegood, J., S. Markx, J. P. Lerch, P. E. Steadman, C. Genç, F. Provenzano, S. A. Kushner, R. M. Henkelman, M. Karayiorgou and J. A. Gogos (2014). "Neuroanatomical phenotypes in a mouse model of the 22q11.2 microdeletion." Mol Psychiatry **19**(1): 99-107.

Feng, S., S. Zheng, H. Zou, L. Dong, H. Zhu, S. Liu, D. Wang, Y. Ning and H. Jia (2022). "Altered functional connectivity of cerebellar networks in first-episode schizophrenia." Front Cell Neurosci **16**: 1024192.

Frascarelli, M., T. Accinni, A. Buzzanca, C. Di Bonaventura, M. Fanella, C. Putotto, B. Marino, M. Pasquini, M. Biondi, C. Colonnese and F. Di Fabio (2023). "Similar grey matter abnormalities in 22q11.2DS and chronic schizophrenia: a voxel-based morphometry study." Rivista di Psichiatria **58**(1): 10-20.

Glickstein, M., P. Strata and J. Voogd (2009). "Cerebellum: history." Neuroscience **162**(3): 549-559.

Gong, D., X. Yu, M. Jiang, C. Li and Z. Wang (2021). "Differential Proteomic Analysis of the Hippocampus in Rats with Neuropathic Pain to Investigate the Use of Electroacupuncture in Relieving Mechanical Allodynia and Cognitive Decline." Neural Plasticity **2021**: 5597163.

Guell, X. and J. Schmahmann (2020). "Cerebellar Functional Anatomy: a Didactic Summary Based on Human fMRI Evidence." Cerebellum **19**(1): 1-5.

Guell, X., J. D. Schmahmann, J. Gabrieli and S. S. Ghosh (2018). "Functional gradients of the cerebellum." Elife **7**.

Gutiérrez, A., Z. U. Khan and A. L. De Blas (1996). "Immunocytochemical localization of the α6 subunit of the γ-aminobutyric acidA receptor in the rat nervous system." Journal of Comparative Neurology **365**(3): 504-510.
Habas, C. (2021). "Functional Connectivity of the Cognitive Cerebellum." Front Syst Neurosci **15**: 642225.
Hanaie, R., I. Mohri, K. Kagitani-Shimono, M. Tachibana, J. Matsuzaki, I. Hirata, F. Nagatani, Y. Watanabe, T. Katayama and M. Taniike (2018). "Aberrant Cerebellar–Cerebral Functional Connectivity in Children and Adolescents With Autism Spectrum Disorder." Frontiers in Human Neuroscience **12**.
Hayes, C., H. Halverson, K. Keeran, K. Tison, K. Jacobo, A. Karki, I. Herring, S. Tunuguntla, M. Pace, B. Doan, H. Wen, A. Klomp, M. Lauffer, M. E. Gaine, K. Parker and A. J. Williams (2023). "16p11.2 Microduplication is Associated with Lobule-Specific Abnormalities in Cerebellar Structure and Function." bioRxiv: 2023.2011.2020.565320.
Hull, C. (2020). "Prediction signals in the cerebellum: Beyond supervised motor learning." eLife **9**: e54073.
Ivry, R. B. and R. M. Spencer (2004). "The neural representation of time." Curr Opin Neurobiol **14**(2): 225-232.
Jackman, S. L., C. H. Chen, H. L. Offermann, I. R. Drew, B. M. Harrison, A. M. Bowman, K. M. Flick, I. Flaquer and W. G. Regehr (2020). "Cerebellar Purkinje cell activity modulates aggressive behavior." eLife **9**: e53229.
Jacobi, H., J. Faber, D. Timmann and T. Klockgether (2021). "Update cerebellum and cognition." J Neurol **268**(10): 3921-3925.
Jones, C. A., D. J. Watson and K. C. Fone (2011). "Animal models of schizophrenia." Br J Pharmacol **164**(4): 1162-1194.
Kim, S. E., S. Jung, G. Sung, M. Bang and S.-H. Lee (2021). "Impaired cerebro-cerebellar white matter connectivity and its associations with cognitive function in patients with schizophrenia." npj Schizophrenia **7**(1): 38.
Kneeland, R. E. and S. H. Fatemi (2013). "Viral infection, inflammation and schizophrenia." Progress in Neuro-Psychopharmacology and Biological Psychiatry **42**: 35-48.
Koziol, L. F. and J. T. Lutz (2013). "From movement to thought: the development of executive function." Appl Neuropsychol Child **2**(2): 104-115.
Lara-Aparicio, S. Y., A. J. Laureani-Fierro, C. Morgado-Valle, L. Beltrán-Parrazal, F. Rojas-Durán, L. I. García, R. Toledo-Cárdenas, M. E. Hernández, J. Manzo and C. A. Pérez (2022). "Latest research on the anatomy and physiology of the cerebellum." Neurology Perspectives **2**(1): 34-46.
Lee, M. T., A. Mouri, H. Kubota, H. J. Lee, M. H. Chang, C. Y. Wu, D. E. Knutson, M. Mihovilovic, J. Cook, W. Sieghart, T. Nabeshima and L. C. Chiou (2022). "Targeting α6GABA(A) receptors as a novel therapy for schizophrenia: A proof-of-concept preclinical study using various animal models." Biomed Pharmacother **150**: 113022.
Magdalon, J., F. Mansur, E. S. A. L. Teles, V. A. de Goes, O. Reiner and A. L. Sertié (2020). "Complement System in Brain Architecture and Neurodevelopmental Disorders." Front Neurosci **14**: 23.
Manto, M.-U. (2003). Chapter 31 Cerebellar ataxias. Handbook of Clinical Neurophysiology. M. Hallett, Elsevier. **1:** 491-520.
Marr, D. (1969). "A theory of cerebellar cortex." J Physiol **202**(2): 437-470.
Meehan, C., L. Harms, J. D. Frost, R. Barreto, J. Todd, U. Schall, C. Shannon Weickert, K. Zavitsanou, P. T. Michie and D. M. Hodgson (2017). "Effects of immune activation during early or

late gestation on schizophrenia-related behaviour in adult rat offspring." Brain Behav Immun **63**: 8-20.
Moberget, T., C. M. Karns, L. Y. Deouell, M. Lindgren, R. T. Knight and R. B. Ivry (2008). "Detecting violations of sensory expectancies following cerebellar degeneration: a mismatch negativity study." Neuropsychologia **46**(10): 2569-2579.
Nakatani-Pawlak, A., K. Yamaguchi, Y. Tatsumi, H. Mizoguchi and Y. Yoneda (2009). "Neonatal phencyclidine treatment in mice induces behavioral, histological and neurochemical abnormalities in adulthood." Biol Pharm Bull **32**(9): 1576-1583.
Nguyen, T. M., L. A. Thomas, J. L. Rhoades, I. Ricchi, X. C. Yuan, A. Sheridan, D. G. C. Hildebrand, J. Funke, W. G. Regehr and W. A. Lee (2023). "Structured cerebellar connectivity supports resilient pattern separation." Nature **613**(7944): 543-549.
Otte, D. M., B. Sommersberg, A. Kudin, C. Guerrero, O. Albayram, M. D. Filiou, P. Frisch, O. Yilmaz, E. Drews, C. W. Turck, A. Bilkei-Gorzó, W. S. Kunz, H. Beck and A. Zimmer (2011). "N-acetyl cysteine treatment rescues cognitive deficits induced by mitochondrial dysfunction in G72/G30 transgenic mice." Neuropsychopharmacology **36**(11): 2233-2243.
Palesi, F., A. De Rinaldis, G. Castellazzi, F. Calamante, N. Muhlert, D. Chard, J. D. Tournier, G. Magenes, E. D'Angelo and C. A. M. Gandini Wheeler-Kingshott (2017). "Contralateral cortico-ponto-cerebellar pathways reconstruction in humans in vivo: implications for reciprocal cerebro-cerebellar structural connectivity in motor and non-motor areas." Sci Rep **7**(1): 12841.
Palesi, F., J. D. Tournier, F. Calamante, N. Muhlert, G. Castellazzi, D. Chard, E. D'Angelo and C. A. Wheeler-Kingshott (2015). "Contralateral cerebello-thalamo-cortical pathways with prominent involvement of associative areas in humans in vivo." Brain Struct Funct **220**(6): 3369-3384.
Raymond, J. L. and J. F. Medina (2018). "Computational Principles of Supervised Learning in the Cerebellum." Annu Rev Neurosci **41**: 233-253.
Reinwald, J. R., A. Sartorius, W. Weber-Fahr, M. Sack, R. Becker, M. Didriksen, T. B. Stensbøl, A. J. Schwarz, A. Meyer-Lindenberg and N. Gass (2020). "Separable neural mechanisms for the pleiotropic association of copy number variants with neuropsychiatric traits." Transl Psychiatry **10**(1): 93.
Rochefort, C., A. Arabo, M. André, B. Poucet, E. Save and L. Rondi-Reig (2011). "Cerebellum shapes hippocampal spatial code." Science **334**(6054): 385-389.
Saadon-Grosman, N., P. A. Angeli, L. M. DiNicola and R. L. Buckner (2022). "A third somatomotor representation in the human cerebellum." Journal of Neurophysiology **128**(4): 1051-1073.
Schiavone, S., P. Tucci, L. Trabace and M. G. Morgese (2019). "Early Celastrol Administration Prevents Ketamine-Induced Psychotic-Like Behavioral Dysfunctions, Oxidative Stress and IL-10 Reduction in The Cerebellum of Adult Mice." Molecules **24**(21): 3993.
Schmahmann, J. D. (2019). "The cerebellum and cognition." Neurosci Lett **688**: 62-75.
Schmahmann, J. D. and D. N. Pandya (1997). "The cerebrocerebellar system." Int Rev Neurobiol **41**: 31-60.
Schmitt, A., P. Falkai and S. Papiol (2023). "Neurodevelopmental disturbances in schizophrenia: evidence from genetic and environmental factors." Journal of Neural Transmission **130**(3): 195-205.
Sendhilnathan, N., M. Semework, M. E. Goldberg and A. E. Ipata (2020). "Neural Correlates of Reinforcement Learning in Mid-lateral Cerebellum." Neuron **106**(1): 188-198.e185.
Sepp, M., K. Leiss, F. Murat, K. Okonechnikov, P. Joshi, E. Leushkin, L. Spänig, N. Mbengue, C. Schneider, J. Schmidt, N. Trost, M. Schauer, P. Khaitovich, S. Lisgo, M. Palkovits, P. Giere, L. M. Kutscher, S. Anders, M. Cardoso-Moreira, ... and H. Kaessmann (2023). "Cellular development and evolution of the mammalian cerebellum." Nature.

Shevelkin, A. V., C. Ihenatu and M. V. Pletnikov (2014). "Pre-clinical models of neurodevelopmental disorders: focus on the cerebellum." Reviews in the Neurosciences **25**(2): 177-194.
Shi, L., S. E. Smith, N. Malkova, D. Tse, Y. Su and P. H. Patterson (2009). "Activation of the maternal immune system alters cerebellar development in the offspring." Brain Behav Immun **23**(1): 116-123.
Silva, N. T., J. Ramírez-Buriticá, D. L. Pritchett and M. R. Carey (2022). "Neural instructive signals for associative cerebellar learning." bioRxiv: 2022.2004.2018.488634.
Stoodley, C. J. and J. D. Schmahmann (2009). "Functional topography in the human cerebellum: a meta-analysis of neuroimaging studies." Neuroimage **44**(2): 489-501.
Stoodley, C. J. and J. D. Schmahmann (2018). "Functional topography of the human cerebellum." Handb Clin Neurol **154**: 59-70.
Stoodley, C. J., E. M. Valera and J. D. Schmahmann (2012). "Functional topography of the cerebellum for motor and cognitive tasks: an fMRI study." Neuroimage **59**(2): 1560-1570.
Strick, P. L., R. P. Dum and J. A. Fiez (2009). "Cerebellum and nonmotor function." Annu Rev Neurosci **32**: 413-434.
Tabata, H., D. Mori, T. Matsuki, K. Yoshizaki, M. Asai, A. Nakayama, N. Ozaki and K.-i. Nagata (2023). "Histological Analysis of a Mouse Model of the 22q11.2 Microdeletion Syndrome." Biomolecules **13**(5): 763.
Tripathi, A., M. Spedding, E. Schenker, M. Didriksen, A. Cressant and T. M. Jay (2020). "Cognition- and circuit-based dysfunction in a mouse model of 22q11.2 microdeletion syndrome: effects of stress." Translational Psychiatry **10**(1): 41.
Uyy, E., V. I. Suica, R. M. Boteanu, F. Safciuc, A. Cerveanu-Hogas, L. Ivan, C. Stavaru, M. Simionescu and F. Antohe (2020). "Diabetic nephropathy associates with deregulation of enzymes involved in kidney sulphur metabolism." J Cell Mol Med **24**(20): 12131-12140.
van Es, D. M., W. van der Zwaag and T. Knapen (2019). "Topographic Maps of Visual Space in the Human Cerebellum." Curr Biol **29**(10): 1689-1694.e1683.
Veleanu, M., B. Urrieta-Chávez, S. M. Sigoillot, M. A. Paul, A. Usardi, K. Iyer, M. Delagrange, J. P. Doyle, N. Heintz, C. Bécamel and F. Selimi (2022). "Modified climbing fiber/Purkinje cell synaptic connectivity in the cerebellum of the neonatal phencyclidine model of schizophrenia." Proceedings of the National Academy of Sciences **119**(21): e2122544119.
Vera-Montecinos, A., J. Galiano-Landeira, M. Roldán, F. Vidal-Domènech, E. Claro and B. Ramos (2023). "A Novel Localization of METTL7A in Bergmann Glial Cells in Human Cerebellum." Int J Mol Sci **24**(9).
Vera-Montecinos, A., R. Rodríguez-Mias, K. S. MacDowell, B. García-Bueno, Á. G. Bris, J. R. Caso, J. Villén and B. Ramos (2021). "Analysis of Molecular Networks in the Cerebellum in Chronic Schizophrenia: Modulation by Early Postnatal Life Stressors in Murine Models." International Journal of Molecular Sciences **22**(18): 10076.
Verpeut, J. L., S. Bergeler, M. Kislin, F. William Townes, U. Klibaite, Z. M. Dhanerawala, A. Hoag, S. Janarthanan, C. Jung, J. Lee, T. J. Pisano, K. M. Seagraves, J. W. Shaevitz and S. S. Wang (2023). "Cerebellar contributions to a brainwide network for flexible behavior in mice." Commun Biol **6**(1): 605.
Wagner, M. J., T. H. Kim, J. Savall, M. J. Schnitzer and L. Luo (2017). "Cerebellar granule cells encode the expectation of reward." Nature **544**(7648): 96-100.
Wang, A.-L., O. Y. Chao, S. Nikolaus, V. Lamounier-Zepter, C. P. Hollenberg, G. Lubec, S. V. Trossbach, C. Korth and J. P. Huston (2022). "Disrupted-in-schizophrenia 1 Protein Misassembly

Impairs Cognitive Flexibility and Social Behaviors in a Transgenic Rat Model." Neuroscience **493**: 41-51.
Wang, C., J. McInnis, M. Ross-Sanchez, P. Shinnick-Gallagher, J. L. Wiley and K. M. Johnson (2001). "Long-term behavioral and neurodegenerative effects of perinatal phencyclidine administration: implications for schizophrenia." Neuroscience **107**(4): 535-550.
Wang, Y., S. Zhong, G. Chen, T. Liu, L. Zhao, Y. Sun, Y. Jia and L. Huang (2018). "Altered cerebellar functional connectivity in remitted bipolar disorder: A resting-state functional magnetic resonance imaging study." Australian & New Zealand Journal of Psychiatry **52**(10): 962-971.
Wilson, E. D., S. R. Anderson, P. Dean and J. Porrill (2019). "Sensorimotor maps can be dynamically calibrated using an adaptive-filter model of the cerebellum." PLoS Comput Biol **15**(7): e1007187.
Yang, J. Z., T. M. Si, Y. Ruan, Y. S. Ling, Y. H. Han, X. L. Wang, M. Zhou, H. Y. Zhang, Q. M. Kong, C. Liu, D. R. Zhang, Y. Q. Yu, S. Z. Liu, G. Z. Ju, L. Shu, D. L. Ma and D. Zhang (2003). "Association study of neuregulin 1 gene with schizophrenia." Mol Psychiatry **8**(7): 706-709.

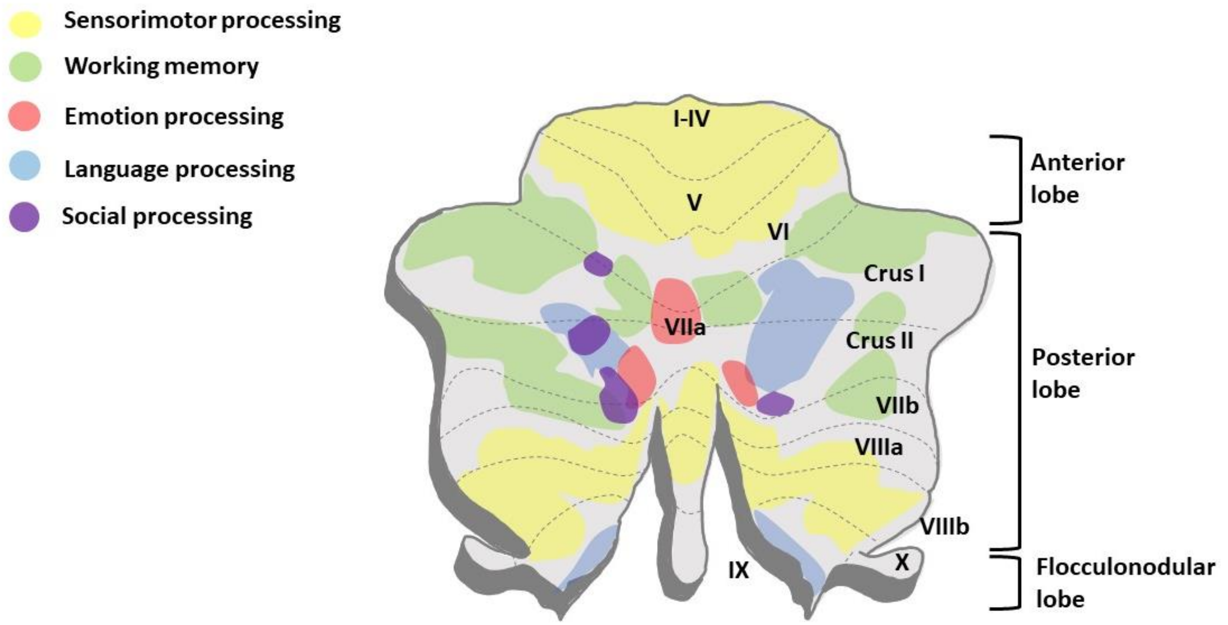

Figure S1: Functional topography of the human cerebellum

The anterior lobe receives connections from the spinal cord and sensorimotor areas and is primarily involved in sensorimotor processes. The posterior vermis receives prominent limbic inputs, and the lobule VI/VII and the corresponding regions in the lateral hemispheres are connected to associative areas of the cerebral cortex (the prefrontal cortex, posterior parietal cortex, cingulate gyrus), involved in higher cognitive functions (Stoodley and Schmahmann 2009) (see (Ciapponi, Li et al. 2023) for an extensive review). The anterior lobe and adjacent parts of lobule VI and the second sensorimotor area in lobule VIII are activated under motor task performance. In contrast, cognitive paradigms activate different regions of the posterior cerebellum. For instance, language and verbal working memory tasks activate the lobule VI and Crus I, executive functions, such as organizing, working memory, or

strategy formation activate the Crus I and lobule VIIB, and finally, tasks involving emotional processing activate vermal lobules VI and VII. However, some tasks are lateralized; for example, language seems to be restricted to the right cerebellum, while spatial functions concern the left cerebellum, which is the opposite of what is seen in the cerebral cortex, implying crossed cerebro-cerebellar projections (Palesi, Tournier et al. 2015, Palesi, De Rinaldis et al. 2017, Guell, Schmahmann et al. 2018). Figure is modified from https://www.the-scientist.com/features/the-multitasking-cerebellum-roles-in-cognition-emotion-and-more-70349 and (van Es, van der Zwaag et al. 2019).
